# Endogenous Expression of HSP70 and Thioredoxin-1 During Methamphetamine-Induced Degeneration

**DOI:** 10.3390/biomedicines14092134

**Published:** 2026-09-21

**Authors:** Michela Ferrucci, Gloria Lazzeri, Maria A. Giambelluca, Paola Lenzi, Carla L. Busceti, Francesco Fornai

**Affiliations:** 1 Human Anatomy, Department of Translational Research and New Technologies in Medicine and Surgery, University of Pisa, 56126 Pisa, Italy; michela.ferrucci@unipi.it (M.F.); gloria.lazzeri@unipi.it (G.L.); maria.giambelluca@unipi.it (M.A.G.); paola.lenzi@unipi.it (P.L.); 2 IRCCS Neuromed, 86077 Pozzilli, Italy; carla.busceti@neuromed.it

**Keywords:** heat-shock proteins, neurodegeneration, neuroprotection, drugs of abuse

## Abstract

**Background/Objectives:** Although HSP70 and thioredoxin-1 (TRX1) are well known to counteract methamphetamine (METH)-induced neurodegeneration, no evidence on the effects of METH administration concerning the level, localization, and co-expression of HSP70 and/or TRX1 is available. Therefore, in the present study, carried out using methamphetamine-treated PC12 cells, we sought to analyze these effects along with the merging of specific markers for mitochondria, autophagy, lysosomes, and chaperone-mediated autophagy (CMA). **Methods**: The levels of specific proteins for specific cell compartments were assessed along with their merging. Thus, co-expression of HSP70 and TRX1 with lipidated LC3 (LC3-II), as well as lysosomal markers such as LAMP1 and LAMP2 (a specific marker for CMA), was assessed. Due to severe mitochondrial alterations produced by METH, both MitoTracker green and MitoTracker red were used in these experimental conditions. Light and electron microscopy, including in situ stoichiometry, were used in these experiments. **Results:** A moderate dose of METH, 100 µM, increases both TRX1 and HSP70; while HSP70 is preferentially co-expressed with the lysosome marker LAMP1, TRX1 is instead co-expressed with the marker LAMP2, which stains for CMA. Both chaperones increase along with cleaved caspase 3 and the autophagosome marker LC3-II. **Conclusions:** The outcome of the present study applies to METH-induced intoxication and is relevant in the fields of neurodegeneration, acute neuronal injury, and the domain of drug abuse. In fact, suppression of TRX1 enables METH-induced addiction, while overexpression of TRX1 or HSP70 produces the opposite effect.

## 1. Introduction

Heat-shock proteins (HSPs) form a large protein family that is highly conserved from plants and bacteria to humans [1,2,3,4,5,6,7,8,9,10,11,12,13]. The name of these proteins comes from their increase in biological tissues following heating, which led to their identification. Later on, overexpression of HSP70 was detected in a number of neurological insults, where it was found to be neuroprotective [14,15,16,17,18,19,20,21]. HSP70 confers neuroprotection against various noxious stimuli [22]. These include acute degenerative conditions (brain ischemia and epilepsy-induced neuronal death) [23,24,25] and chronic degenerative disorders such as Parkinson’s disease (PD) [26,27,28,29,30,31,32,33] and Alzheimer’s disease (AD) [33,34,35]. Remarkably, a strong protective effect of HSP70 was reported in experimental models featuring neurotoxicity related to alpha-synuclein aggregation [36,37,38], including neurodegeneration produced by the abuse of psychostimulants such as methamphetamine (METH) [39]. In detail, HSP70 protects against METH-induced neurotoxicity/neurodegeneration [40,41,42]. These effects recruit cell-clearing systems, which are engaged by HSP70 [43,44,45]. This was recently established by measuring the presence of HSP70 within autophagic vacuoles and its concomitance with microtubule-associated protein 1A/1B-light chain 3 (LC3) [43]. In line with this, METH triggers molecules involved in cell clearance, as shown by poly-ubiquitin and p62-positive neuronal inclusions along with some alpha-synuclein aggregates within tubule-vesicular structures [39,46,47,48,49,50,51,52,53,54,55,56,57,58]. This cytopathology is reminiscent of that described during neurodegenerative disorders characterized at first by ubiquitin aggregates [51,52,53]. By incidence, ubiquitin is seminal in starting cell clearance, and it is a member of the HSP family [54,55]. Similarly to HSP70, ubiquitin is strongly induced under acute and chronic degenerative conditions [39,52]. In detail, the expression of ubiquitin and its binding to altered substrates is key to delivering these cargos to the proteasome (attached to poly-ubiquitin chains) [56,57,58] and autophagy (attached to either mono- or poly-ubiquitin chains) [49,59,60,61,62]. In line with a protective role, the activation of cell clearance (either through the proteasome or autophagy or both) protects against METH toxicity [52,63,64,65,66,67,68]. This is in line with the findings that HSP70 overexpression protects against METH-induced aggregation of alpha-synuclein [41]. Despite the evidence about the protective effects of HSP70 against METH toxicity to catecholamine cells being well established, no study so far has analyzed whether administration of METH does indeed elevate endogenous HSP70 within catecholamine neurons. This is highly relevant considering that in humans who abuse METH, HSP70 increases in a number of peripheral organs [69]. Due to its effects in delivering toxic cargos to neutralize their toxicity, HSP70 behaves as a chaperone [70,71,72,73,74,75,76], which produces its effects through the interaction with other proteins (so-called co-chaperones) [77]. A seminal event, which is critical in the mechanism of action of HSP70, consists of the interaction with thioredoxin-1 (TRX1) [78]; this latter protein, like HSP70, is induced following heat shock [79], and it possesses co-chaperone activity [80,81,82]. Thioredoxin-1 exerts both antioxidant and co-chaperone effects [83,84,85,86,87,88], and, similarly to HSP70, protects against MPTP toxicity [89,90,91,92,93,94] and METH toxicity [95,96,97,98,99]. These studies report that increasing or decreasing the expression of this protein, just like for HSP70, leads either to protect or worsen, respectively, METH-induced cell damage. Despite their protective role being well established, no evidence is available showing whether METH administration produces a natural upregulation of endogenous HSP70 and/or TRX1. Therefore, in the present study, we sought to assess whether METH alters the level of HSP70 or natural TRX1 spontaneously expressed in the absence of genetic manipulation. When this occurs, it would be important to assess whether hypothetical endogenous up- (or down-) regulation of TRX1 is concomitant with HSP70 up- (or down-) regulation. Again, it is crucial to establish whether, following METH administration, a hypothetical endogenous overexpression of HSP70 and TRX1 is concomitant; whether this occurs widely in the cytosol; and whether it involves specific cell compartments. Again, proteins being co-expressed with HSP70 or TRX1 may help identify cell compartments that are relevant to counteract the molecular mechanisms of degeneration.

In the present study, expression of either HSP70 or TRX1 was calculated following various doses of METH. This follows a dose–response study that was carried out to assess METH-induced cell death and degeneration.

Once a moderate dose of METH was selected, the expression of HSP70 and TRX1 was assessed. The merging of HSP70 and TRX1 was assessed using light microscopy and further analyzed by in situ stoichiometry and electron microscopy. Due to the potential involvement of specific organelles in neurodegeneration, the expression of HSP70 and TRX1 was assessed in combination with lipidated LC3 (LC3-II) as a marker of activated autophagosomes. Similarly, the lysosomal-associated membrane protein 1, LAMP1, and lysosomal-associated membrane protein 2, LAMP2 (a specific marker of lysosomes and chaperone-mediated autophagy, CMA, respectively [100,101,102,103,104,105,106,107,108,109,110,111,112]) were measured. Protein merging was aimed at establishing which cell organelle was engaged by these proteins. Due to severe alterations produced by METH at the mitochondrial level, both MitoTracker green (MTR-G, staining all mitochondria) and MitoTracker red (MTR-R, staining healthy mitochondria) were used. Mitochondrial integrity was related to the level of mitochondrial localization of either HSP70 or TRX1, and mitochondrial status was established using ultrastructural morphometry.

The outcome of the present study applies to METH-induced intoxication, and it is relevant to the fields of neurodegeneration, acute neuronal injury, and the domain of drug abuse [113,114,115,116,117,118,119]. In fact, it was recently demonstrated that suppression of TRX1 enables METH-induced addiction [95,96], while overexpression of TRX1 or HSP70 produces the opposite effect [41]. Again, compounds such as geranylgeranylacetone (GGA), which act as inducers of both TRX1 and HSP70, protect against METH-induced degeneration [40].

## 2. Materials and Methods

### 2.1. Cell Cultures

The pheochromocytoma PC12 cell line, purchased from a cell bank (IRCCS San Martino Institute, Genova, Italy), was grown in RPMI 1640 medium (Sigma-Aldrich, St. Louis, MO, USA), supplemented with heat-inactivated 10% horse serum (HS, Sigma-Aldrich), 5% fetal bovine serum (FBS, Sigma, St. Louis, MO, USA), and penicillin (50 IU/mL)/streptomycin (100 µg/mL, from a stock solution of 50 mg/mL, Sigma-Aldrich). Cells were kept under standard conditions in a humidified atmosphere with 5% CO_2_ at 37 °C. All experiments were carried out during the log-phase of cell growth, corresponding to approximately 70% confluence [120,121].

The rationale for the use of undifferentiated PC12 cells is based on their widespread use in the neurobiological literature to study the effects of METH on catecholamine cells, compared with differentiated PC12 cells. Indeed, when exposed to differentiating agents, together with acquisition of a neuron-like phenotype, PC12 cells might modify their typical neurochemical profile, resulting in a reduction in catecholamine synthesis and production of acetylcholine [122,123].

For the light microscopy experiments, 5 × 10^4^ cells were seeded on poly-lysine-coated coverslips, which were placed in 24-well plates with 1 mL/well of culture medium.

For the electron microscopy experiments, 1 × 10^6^ PC12 cells were seeded in 6-well plates with 2 mL/well of culture medium.

### 2.2. Cell Treatments

A METH stock solution was prepared by dissolving 2.3 mg of METH in 1 mL of culture medium. Working solutions were obtained by diluting appropriate aliquots of the stock solution in the culture medium to reach the final concentrations.

For dose–response experiments aimed at evaluating cell survival, PC12 cells were exposed to increasing concentrations of METH, ranging from 10 µM to 1000 µM, for 72 h. These experiments confirmed the findings of our previous studies, which demonstrated that exposure to 100 µM METH for 72 h induced moderate cell death [50,124,125,126,127,128]. This condition was therefore selected for subsequent experiments to investigate specific protein expression under moderate METH-induced toxicity.

In the experiments aimed at assessing autophagic flux, the autophagy inhibitor bafilomycin A1 (100 nM, Sigma-Aldrich) was added to the culture medium 3 h before the end of the treatments with METH.

Control cells were maintained in the same volume of culture medium for the same duration. At the end of the treatment period, the culture medium was removed, and cells were washed with PBS and processed according to the specific experimental procedures.

### 2.3. Hematoxylin and Eosin (H&E) Staining

After fixation with 4% paraformaldehyde for 15 min, PC12 cells were washed in PBS and then immersed in the hematoxylin solution (Sigma-Aldrich) for a few minutes. The hematoxylin staining was stopped by repeated washing with running water. Then, cells were submerged in an eosin solution (Sigma-Aldrich) for a few seconds and washed out again to remove the excess dye. After dehydration in increasing concentrations of alcohol, cells were cleared using xylene, gently transferred to a slide, and covered with DPX mounting medium (Sigma-Aldrich).

Slides were observed under a Nikon Eclipse Ni light microscope (Nikon, Tokyo, Japan), equipped with a digital camera connected to the NIS Elements software (NIS Elements D 5.30.00 build 1531, 64-bit) for image analysis.

### 2.4. Fluoro-Jade B (FJ-B) Histofluorescence

At the end of the treatments, the medium was removed, and cells were washed in PBS and immersed in a fixing solution containing 4% paraformaldehyde in PBS for 5 min. After a quick wash in PBS, cells were incubated in a 0.06% potassium permanganate solution for 10 min at room temperature (RT). Then, cells were washed in distilled water and incubated for 20 min in a 0.0004% FJ-B solution. This solution was prepared by diluting a 0.01% FJ-B stock solution (Merck Millipore, Billerica, MA, USA) in distilled water containing 0.1% acetic acid. After repeated washing in PBS, cells were gently transferred to a slide; then, they were finally covered with DPX mounting medium (Sigma-Aldrich) and analyzed under a Nikon Eclipse Ni light microscope (Nikon, Tokyo, Japan), which was equipped with a fluorescence lamp and a digital camera connected to the NIS Elements software (NIS Elements D 5.30.00 build 1531, 64-bit) for image analysis (Nikon, Tokyo, Japan).

### 2.5. MitoTracker Red (MTR-R) and MitoTracker Green (MTR-G) Histofluorescence

To stain mitochondria in living cells, the red fluorescent dye MTR-R, which labels healthy mitochondria, and the green fluorescent dye MTR-G, which labels total (both healthy and unhealthy) cell mitochondria, were used [127,129,130,131]. In detail, cells were incubated in a solution of MTR-R (Thermo-Fisher Scientific, Waltham, MA, USA) or MTR-G (Thermo-Fisher Scientific) at 500 nM in a serum-free culture medium for 45 min at 37 °C and 5% CO_2_. At the end of incubation, the MTR-R or MTR-G solution was removed, and fresh, pre-warmed medium was added. Cells were immediately analyzed under a Nikon Eclipse Ni microscope, equipped with a fluorescent lamp and a digital camera connected to the NIS Elements software (NIS-Elements D 5.30.00 build 1531, 64-bit) for image analysis (Nikon, Tokyo, Japan).

### 2.6. Immunofluorescence

At the end of the treatments, the medium was removed, cells were washed in PBS, and then they were immersed in the fixing solution, consisting of 4% paraformaldehyde in PBS, for 15 min. After fixation, cells were washed in PBS and submerged in a PBS solution of 0.1% Triton X-100 (Sigma-Aldrich) for 15 min, washed again in PBS, and immersed in a blocking solution containing 10% normal goat serum in PBS for 1 h at RT, which allowed for saturating the non-specific binding sites. Cells were then incubated overnight at 4 °C in 1% normal goat serum in PBS containing the following primary antibodies:

(i) anti-HSP70 antibody (Abcam, Cambridge, UK); (ii) anti-TRX1 antibody (R&D Systems, Minneapolis, MN, USA); (iii) anti-cleaved caspase 3 antibody (Cell Signaling Technology, Danvers, MA, USA); (iv) anti-LC3-II antibody (Cell Signaling Technology); (v) anti-LAMP1 antibody (Abcam); (vi) anti-LAMP2 antibody (Abcam); (vii) anti-p62 antibody (Invitrogen, Carlsbad, CA, USA).

All primary antibodies were diluted 1:100. After washing in PBS, cells were incubated for 1 h with the appropriate fluorophore-conjugated secondary antibodies, consisting of Alexa488 or Alexa546 (Life Technologies, Carlsbad, CA, USA). All secondary antibodies were diluted 1:200. After washing in PBS, cell nuclei were stained using the fluorescent dye DAPI (Sigma-Aldrich) diluted 1:1000 in distilled water. Then, cells were washed again in distilled water, gently transferred to a slide, and then they were finally covered with the mounting medium Fluoro-shield (Sigma-Aldrich). Slides were observed using a Nikon Eclipse Ni light microscope, which was equipped with a fluorescent lamp and a digital camera connected to the NIS Elements software (NIS-Elements D 5.30.00 build 1531, 64-bit) for image analysis (Nikon, Tokyo, Japan). Control sections were incubated with secondary antibodies only.

### 2.7. Transmission Electron Microscopy (TEM)

Cells were centrifuged at 1000× *g* for 5 min and rinsed in PBS. Then, cell pellets were submerged in a first fixing solution (2.0% paraformaldehyde and 0.1% glutaraldehyde in 0.1 M PBS, pH 7.4) for 90 min at 4 °C. After three rounds of PBS washing for 5 min each time, cells were fixed in a second fixing solution (1% osmium tetroxide, OsO_4_, in PBS) for 1 h at 4 °C. Then, cells were dehydrated using increasing concentrations of ethanol (from 30% up to 100%) and left in propylene oxide for 1 h before finally being embedded in epoxy resin. This protocol was set up based on our previous investigations, aimed at optimizing sample preparation for both plain and immunogold-based TEM [128]. The first fixing solution consisted of moderate concentrations of two aldehydes, which was intended to expose—as much as possible—the binding sites of antigens (to be stained with immunogold), while the second fixing solution was used to produce a remarkable contrast useful for distinguishing membrane-bound cell organelles and fine intracellular trim (to perform the in situ analysis of various cell domains).

Ultrathin slices (90 nm) were used for both plain and immunogold-based electron microscopy. Ultrathin slices were stained with uranyl acetate and lead citrate, and were observed using a JEOL JEM SX100 electron microscope (JEOL, Tokyo, Japan).

### 2.8. Post-Embedding Immunogold Electron Microscopy

Ultrathin slices collected on nickel grids were placed for 30 min on aqueous sodium metaperiodate droplets, at RT, to remove OsO_4_ and unmask antigen epitopes [132,133]. After repeated washing in PBS, grids were placed on droplets of a PBS 1 M blocking solution (10% goat serum and 0.2% saponin) for 20 min at RT to prevent non-specific staining. Then, grids were incubated at 4 °C overnight on droplets of the primary antibody solution, which consisted of primary antibodies diluted in ice-cold PBS solution with 1% goat serum and 0.2% saponin to increase antigen binding. In detail, the primary antibodies used were as follows: (i) anti-HSP70 antibody (Abcam), diluted 1:50; (ii) anti-TRX1 antibody (R&D Systems), diluted 1:50.

At the end of the incubation, grids were washed in cold PBS (three times, 5 min each) and were placed for 1 h at RT on droplets of a PBS solution containing 1% goat serum, 0.2% saponin, and gold-conjugated (10 nm or 20 nm gold particles) secondary antibodies (BB International, Cardiff, UK), diluted 1:100.

After washing in PBS (three times, 5 min each), grids were placed for 3 min on droplets containing 1% glutaraldehyde dissolved in PBS. Then, they were washed in distilled water and stained with uranyl acetate and lead citrate. Sliced ultrathin sections were finally observed on TEM (JEOL JEM SX100). Control ultrathin sections were incubated with secondary antibodies only.

Counts on TEM were carried out using a magnification of 8000×, which allows for immunogold detection within specific subcellular compartments.

Ultrastructural morphometry on conventional TEM was performed to quantify organelles (vacuoles and mitochondria). Mitochondrial alterations were defined as follows, in accordance with our previous studies [131]: (i) reduced matrix electron density (dilution, vacuolization, and presence of electron-lucent foci); (ii) fragmented, disorganized, and ballooned cristae (intra-cristal swelling); (iii) partial or total separation of the outer and inner mitochondrial membranes; (iv) presence of dense, osmiophilic laminated or whorled membranes within mitochondria; (v) mitochondrial swelling. To be included in the group of altered mitochondria, each mitochondrion was required to display at least two alterations.

The site specificity of immunogold particles against HSP70 or TRX1, assessed through in situ stoichiometry [133], made it possible to quantify the actual amount of each protein within specific subcellular compartments. This approach enabled the assessment of changes in specifically stained vacuoles or mitochondria under METH treatment compared with controls. The total amount of each protein within the cytosol and within discrete organelles was measured, and the effects of METH were evaluated by determining the increase or decrease in the protein both in the cytosol and within vacuoles or mitochondria. This allowed us to infer how protein compartmentalization was modified under METH exposure.

### 2.9. Extended Statistics

#### 2.9.1. Light Microscopy

To document cell viability, quantitative measurements of the number of H&E- and FJ-B-positive cells were carried out under light microscopy using 3 different microscopic fields from each experimental group, visualized at 20× magnification, allowing the analysis of an area of about 6.3 × 10^5^ µm^2^. Each field was randomly selected from each experimental group. Cell counts were carried out by two researchers who were blind to treatments. In this way, a decrease in the cell number was detectable following METH.

Instead, when measurements of antigen expression or organelle markers were carried out, mean fluorescence intensity and area per cell were calculated considering the mean ± S.E.M. of 70 cells for each treatment, which were randomly chosen at 40× magnification. These cells were chosen independent of their occurrence within a cluster or their occurrence as isolated cells within the slide.

Again, since cell clusters occur quite regularly throughout the slide, the random selection guarantees a valid representation of cell distribution, thus avoiding bias in the sampling procedure (which may occur when focusing only on cell clusters, isolated cells, or empty areas of the slide). Random selection is the only way to avoid unbalanced counts in the slide and provide a true representation of spatial distribution of cell density. Each criterion leading to select specific areas in the slide would introduce a bias in data selection. The measurement of fluorescent intensity and fluorescent areas was carried out using light microscopy from a total of *n* = 70 cells, which were randomly analyzed within 3 microscopic fields at 40× magnification. This is critical considering that the fluorescence intensity and fluorescent area of each cell may vary depending on whether the cell occurs within a cluster or is isolated. Indeed, in this sampling process we also noted that within each treatment group, the amount of clustering of the cells does not influence the intensity and the area of fluorescence within the cells themselves. Since immunofluorescence data refer to the mean fluorescence intensity and mean fluorescent area within a single cell, when 70 samples are counted from each group, they provide high statistical power. This is further validated by the very low variability between various cells from the same group. Immunofluorescent areas were measured under a fluorescence microscope using the software ImageJ (NIH, Version 1.8.0_172, Bethesda, MD, USA). For merging immunofluorescence, a novel index was introduced here to emphasize the specificity and cell focality of merging either HSP70 or TRX1 with compartment-specific proteins. This was called the specificity index. Remarkably, the highest ratio was observed between the merging of a given protein with a housekeeping protein marker of a cell compartment and the increase in that protein in whole cells—the highest being its compartmentalization. In other words, the paucity of cell sites where the protein increases and the net amount of such a focal increase express the specificity of a protein increase within a specific localization. This applies to the specificity of either TRX1 or HSP70 within LC3-II-positive autophagosomes, LAMP1-positive lysosomes, or LAMP2-positive organelles where CMA is supposed to occur.

Values are given as the mean percentage ± S.E.M. of H&E-stained cells (assuming controls = 100%) or, alternatively, the authentic mean number ± S.E.M. of FJ-B-fluorescent cells following each treatment. Each experiment was carried out in triplicate.

Densitometry was measured based on a cut-off concerning optical density according to Marwaha and Sharma [134].

Comparisons between experimental groups were calculated using one-way ANOVA with Sheffe’s post hoc analysis. The null hypothesis was rejected at *p* < 0.05.

#### 2.9.2. Transmission Electron Microscopy

Antigen detection was further supported and confirmed by the count of 50 cells per group, where antigen detection was revealed by immunogold TEM, which produced a quantitative value for the amount of each antigen under analysis. In the immunogold counts, TEM was used to examine non-serial ultrathin slices (90 nm thick) at a distance of 10 µm, which avoided counting the same cell twice. Cells were counted at 8000× magnification on 2–3 slices collected on different grids (to further avoid bias brought by the mechanical support). Since the trimming of the embedded sample substantially reduces the surface being cut, ultrathin slices usually contain roughly 25 cells.

Cells counts included the followings: (i) the number of HSP70 or TRX1 immunogold particles placed within the cytosol; (ii) the number HSP70 or TRX1 immunogold particles within the vacuoles; (iii) the number HSP70 or TRX1 immunogold particles within the mitochondria; (iv) the number of HSP70 or TRX1 immuno-positive vacuoles; (v) the number of vacuoles per cell; (vi) the number of mitochondria per cell; (vii) the number of altered mitochondria per cell; (viii) the number of healthy mitochondria per cell; (ix) both HSP70- + TRX1-positive vacuoles; (x) both HSP70- + TRX1-positive mitochondria; (xi) an increase or decrease in the protein (HSP70 or TRX1) ratio between vacuoles or mitochondria and cytosol. Each value was reported as the mean ± S.E.M. of 50 cells per group. The total number of mitochondria/vacuoles in the whole cell greatly exceeds the number counted within a TEM ultrathin slice, which rather covers the surface of less than 5% of the whole cell. Considering a cell diameter of 20 µm and the slice thickness of 90 nm, the number of mitochondria in each slice needs to be multiplied by 222.22, which yields a total number of over 5000, which corresponds to the general counts reported for the number of mitochondria in whole cells. The same consideration needs to be applied to the count of vacuoles. Statistical comparisons were performed using one-way ANOVA with Sheffe’s post hoc analysis. The null hypothesis was rejected at *p* < 0.05.

## 3. Results

### 3.1. METH Produces Dose-Dependent and Severe Damage to Catecholamine Cells

#### 3.1.1. H&E

**Figure 1:**
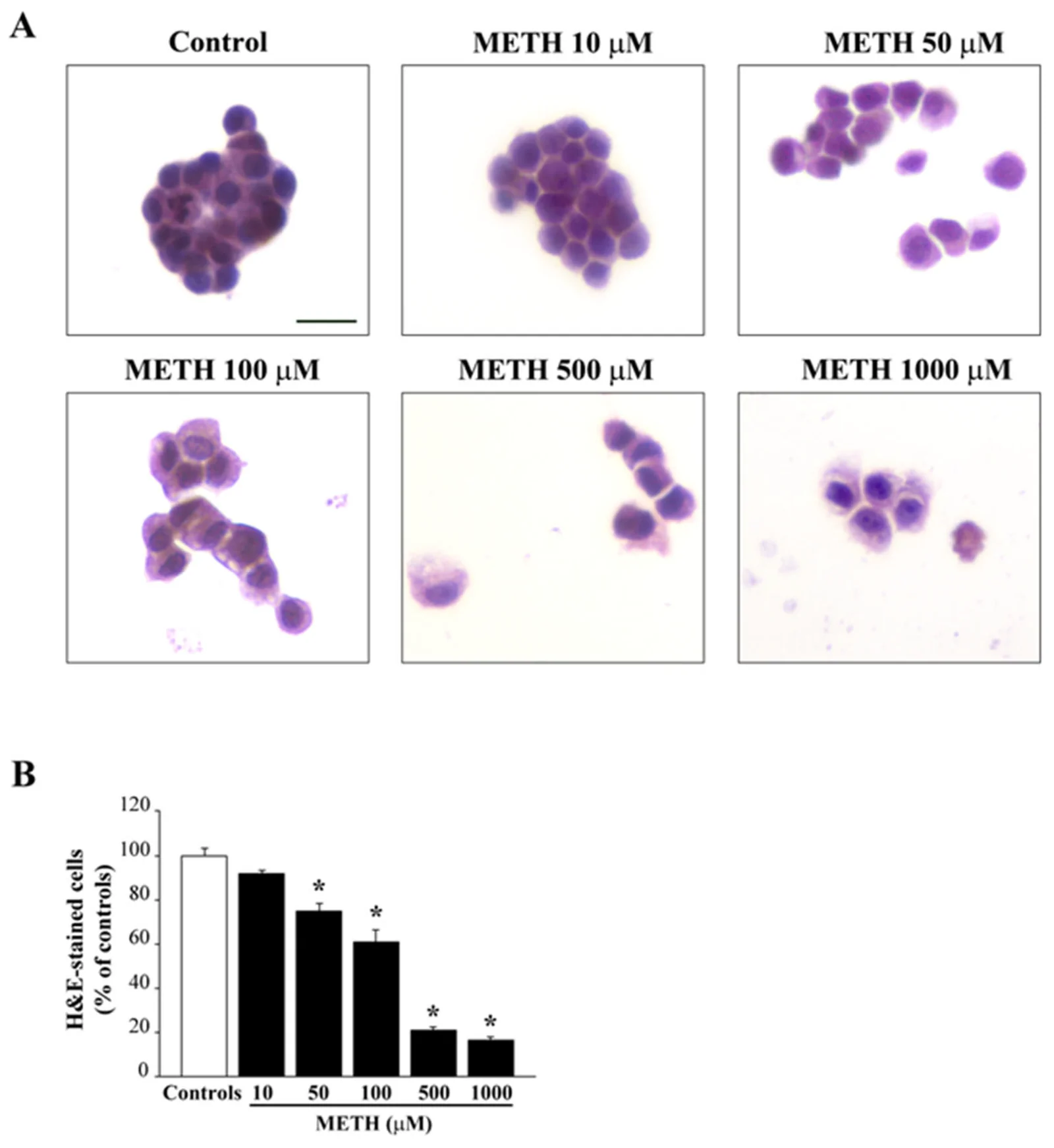
METH produces a dose-dependent loss of cell viability, as assessed by H&E staining. Representative pictures in (**A**) report the progressive effects of METH. The graph in (**B**) reports values for cell viability, which decreases dose-dependently as a percentage of controls (considering controls = 100%), starting from the METH dose of 50 µM. Each count represents the percentage mean ± S.E.M. of H&E-stained cells counted under 20× magnification within three microscopic fields, where only distinct cells that were not overlapping were considered in the count. Each experiment was carried out in triplicate. * *p* < 0.05 compared with controls. Scale bar (**A**) = 24 µm.

Representative pictures of H&E-stained PC12 cells are shown in Figure 1A. Control cells appear small in size, round in shape, with intensely hematoxylin-stained nuclei and a homogeneously stained cytosol. After treatment with increasing doses of METH, ranging from 10 µM up to 1000 µM, cells appear with an abnormal, no longer roundish shape and a smaller size, where the cytosol appears crowded with irregular eosinophilic areas surrounded by pale vesicle-like domains (Figure 1A). These dose-dependent pathological changes are reminiscent of the tubulo-vesicular structures that were recently described in vitro in the course of METH-induced cell degeneration [125]. These areas were recently investigated by combined light and electron microscopy (CLEM) as pale eosinophilic, non-electron-dense cell domains that recruit components of the autophagolysosomal pathway, with several damaged mitochondria, high concentrations of p62, and the small heat-shock protein ubiquitin, along with some amount of alpha-synuclein [49,50]. The histochemical staining with H&E allows for counting the dose-dependent decrease in cell number (see the graph in Figure 1B).

#### 3.1.2. Cleaved Caspase 3

**Figure 2:**
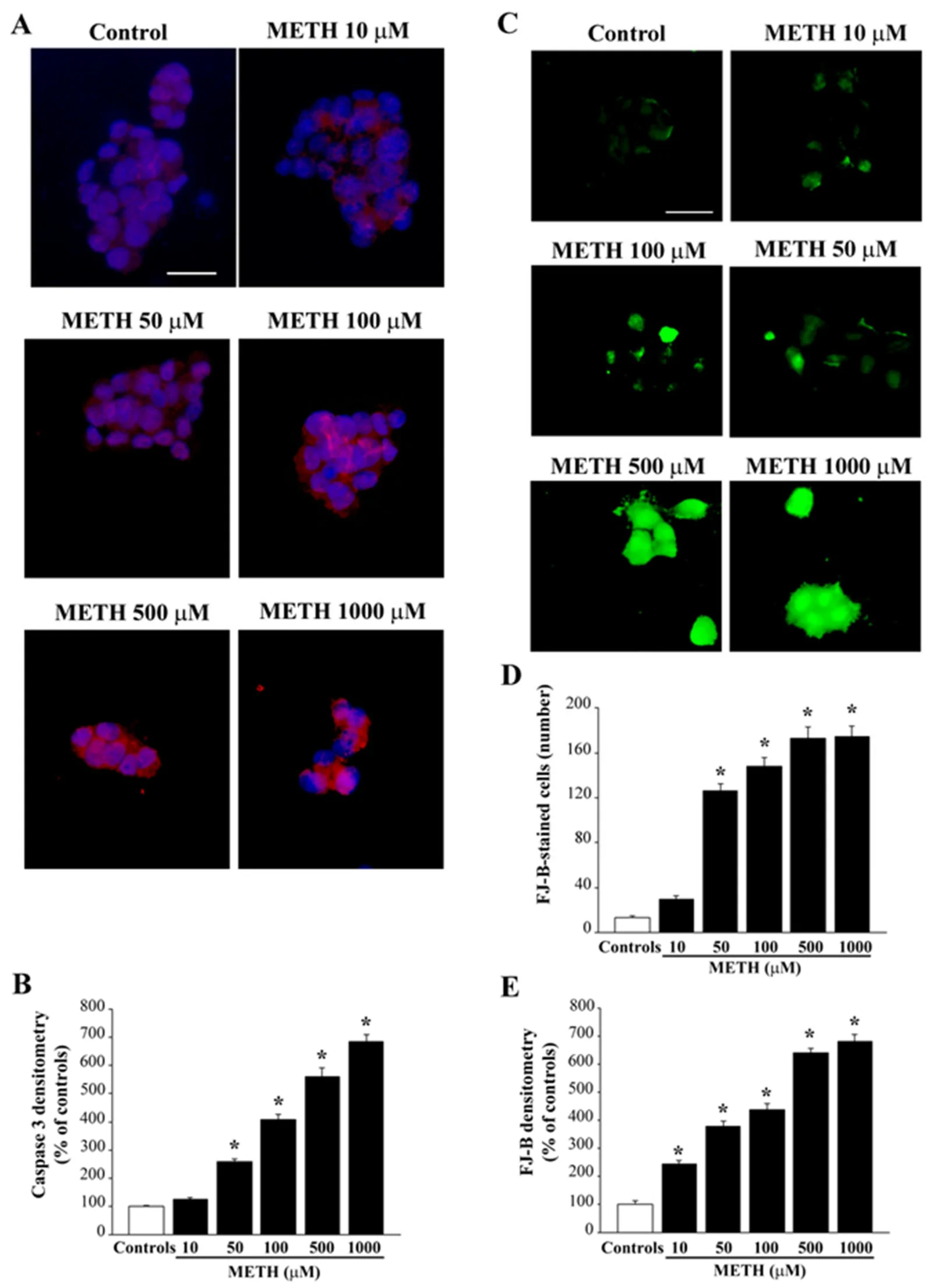
Immunofluorescence for caspase 3 and histofluorescence for FJ-B increase dose-dependently following METH. Representative pictures in (**A**) show cell fluorescence for cleaved caspase 3 following various doses of METH. The increase in caspase 3 immunofluorescence was progressive, according to a quasi-linear distribution. Such a linear progression is displayed in graph (**B**), reporting the mean percentage ± S.E.M. of densitometry measured in each experimental group (*n* = 70), considering controls = 100%. Representative pictures in (**C**) display fluorescent staining of cell death using the degeneration-specific marker FJ-B. FJ-B fluorescent cells, which are scarcely detectable in control conditions, increase progressively to reach a plateau at 500 µM METH. Counts of the number of FJ-B fluorescent cells are reported in graph (**D**), while cell densitometry is reported in graph (**E**). The counts of FJ-B fluorescent cells are expressed as the mean ± S.E.M. of FJ-B-stained cells counted under 20× magnification within three microscopic fields. Each experiment was carried out in triplicate. Densitometric measurement of caspase-3- or FJ-B-positive cells is reported as the mean percentage ± S.E.M. from *n* = 70 cells (assuming controls = 100%). * *p* < 0.05 compared with controls. Scale bars (**A**) = 24 µm; (**C**) = 20 µm.

The occurrence of cell degeneration assessed by H&E is indicated by a decrease in cell number, as reported in the graph in Figure 1B. The dose-dependent cytopathology following various METH doses was better expressed by the quasi-linear distribution of cell fluorescence observed following fluorophore-conjugated secondary antibodies binding anti-cleaved caspase 3 primary antibodies. This immunostaining provided an increase in single-cell fluorescence from 10 µM METH up to 1000 µM METH, as shown in the representative pictures in Figure 2A. Such an increase was calculated according to immunofluorescent densitometry, as reported in the graph in Figure 2B. It is evident that the dose producing moderate degenerative effects corresponds to 100 µM METH.

This procedure applied to METH toxicity requires some theoretical considerations. The routine non-critical application of cleaved caspase 3 as synonymous with cell death may be somewhat misleading since cleaved caspase 3 may be induced even by strong cell activity in the absence of cell death [135,136,137,138,139]. In keeping with this, it is well known that caspase 3 may contribute to counteracting neurodegeneration and aggregation of proteins in neurodegeneration. Since the occurrence of apoptosis does not appear in the TEM following METH, it is likely that the measurement of cleaved caspase 3 reflects more of a compensatory mechanism rather than having a detrimental pro-apoptotic effect. This is why the mere assessment of cleaved caspase 3 expression was definitely not enough to validate the loss of cell viability as an add-on procedure to H&E histochemistry. Therefore, we selected a rougher, less articulated marker of cell degeneration without secondary implications, consisting of FJ-B histochemistry. Within this scenario, cleaved caspase 3 may be challenged for merging with HSP70 on the same cells (see below) to probe the role of caspase beyond a marker of cell degeneration, rather as a marker of protein aggregation.

#### 3.1.3. FJ-B

Therefore, to implement fluorescent staining of cell death, the degeneration-specific marker FJ-B was used in addition (see the representative pictures in Figure 2C). It is evident that the fluorescence becomes increasingly intense as the dose of METH increases up to 1000 µM. This is due to the fact that the yellow fluorescent dye FJ-B binds polyamines accumulating within degenerating cells (Figure 2C). As shown in the representative pictures in Figure 2C, FJ-B fluorescent cells—which are scarcely detectable in control conditions—increase progressively in a dose-dependent manner following METH (Figure 2C). Counts of the number of FJ-B fluorescent cells, which are reported in the graph in Figure 2D, confirm that METH administration dose-dependently causes cell degeneration, starting at 50 µM. It is remarkable that fluorescent areas following cleaved caspase 3 diverge from the fluorescent area following FJ-B, suggesting that the significance of caspase 3 goes beyond the staining of degenerating areas.

It needs to be clarified that increased staining for these fluorescent markers of neurodegeneration occurs at the single-cell level. If one considers the total cell population, a marked decrease in the number of cells is predominant. Such a decrease in cell density is massively occurring up to 1000 µM, and the number of cell loss could be counted directly by H&E. This avoids the bias provided by an increase in histofluorescent cells with FJ-B being interpreted as an increase in cell density (see the graph in Figure 2C). The measurement of the mean fluorescence per cell (see the graph in Figure 2E) is much more reliable as an index of neurodegeneration. This confirms what was reported previously, following H&E by the graph in Figure 1B, which measures a dose-dependent decrease in cell number starting at the METH dose of 50 µM.

### 3.2. METH Induces a Dose-Dependent Increase in HSP70

**Figure 3:**
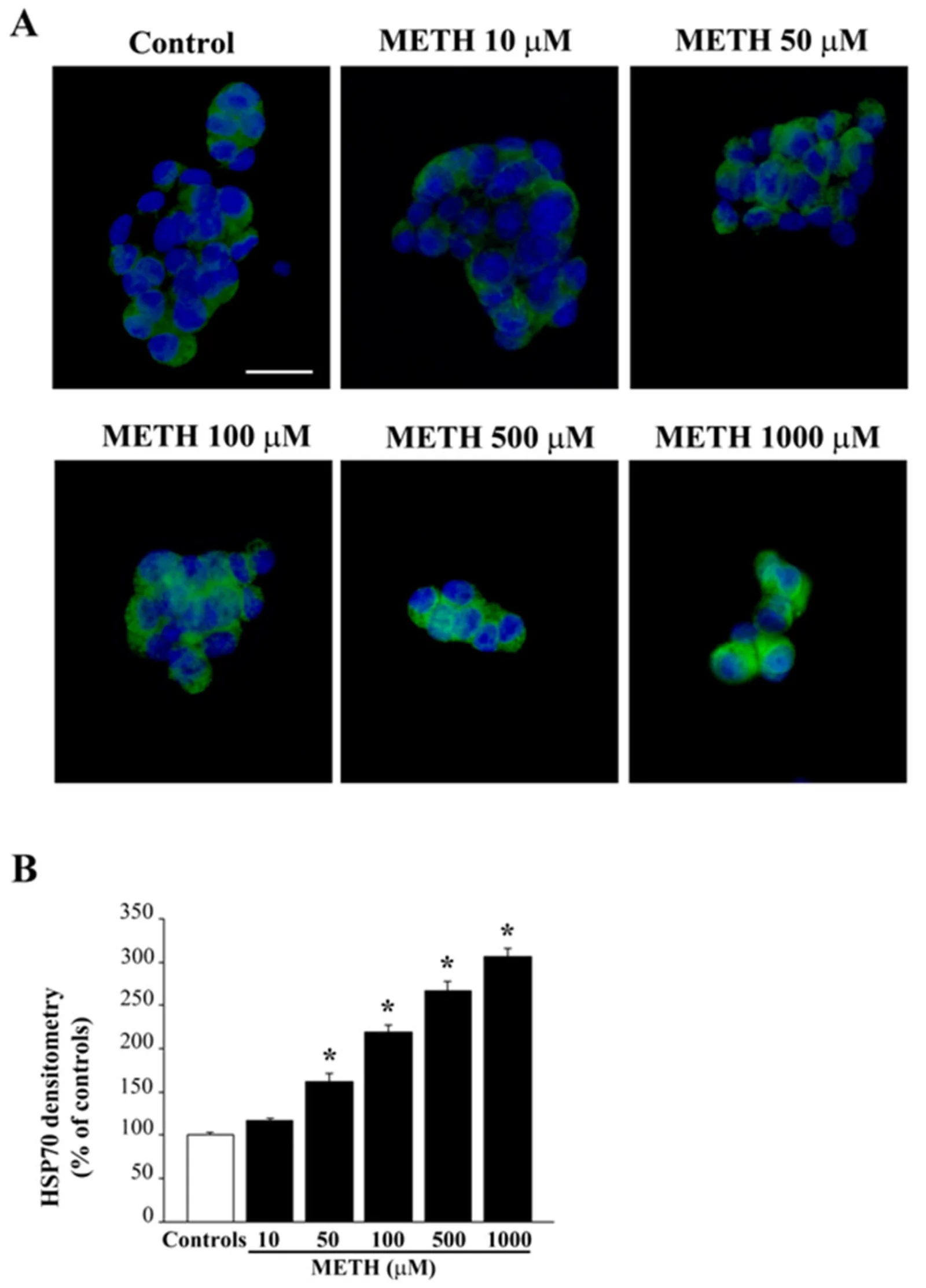
Immunofluorescence for HSP70 increases dose-dependently following METH. Representative pictures in (**A**) show the increase in HSP70 immunofluorescence produced by increasing doses of METH. Since HSP70 protects against METH toxicity, this indicates that METH administration may trigger an increase in HSP70 as a compensatory effect. Data reported in graph (**B**) were obtained by measuring densitometry from *n* = 70 cells per group. Data are reported as the mean percentage ± S.E.M., assuming controls = 100%. * *p* < 0.05 compared with controls. Scale bar (**A**) = 18 µm.

The increase in cell damage produced by increasing doses of METH triggered a concomitant increase in HSP70 (see the representative pictures in Figure 3A and the graph in Figure 3B), which protects against METH toxicity [41]. This indicates that METH administration may trigger an increase in HSP70 as a compensatory effect.

**Figure 4:**
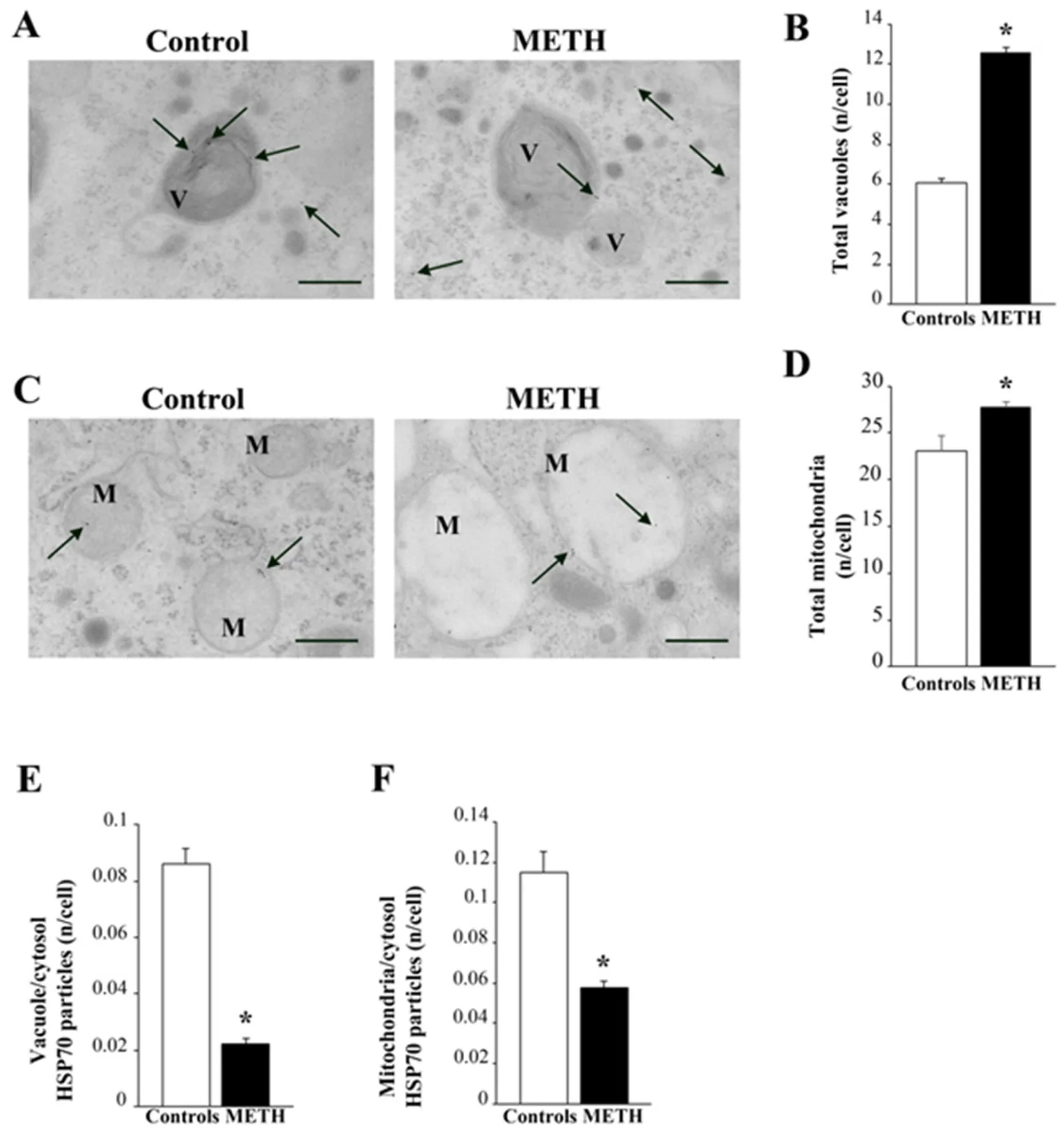
METH alters cellular localization of HSP70. Representative micrographs in (**A**) show vacuoles stained with HSP70 immunogold particles (arrows) within controls or cells treated with 100 µM METH. The graph in (**B**) reports the total number of vacuoles within controls and METH-treated cells. Representative micrographs in (**C**) show mitochondria stained with HSP70 immunogold particles (arrows), while graph in (**D**) reports the total number of mitochondria. The graphs show (**E**) the ratio between vacuole and cytosol HSP70 and (**F**) the ratio between mitochondria and cytosol HSP70. Each value represents the mean ± S.E.M. of *n* = 50 cells per group. * *p* < 0.05 compared with controls. V = vacuole; M = mitochondria. Scale bars (**A**,**C**) = 400 nm.

Since the silencing of HSP70 worsens METH toxicity and overexpression of HSP70 protects against METH toxicity [41], the progressive increase in the endogenous protein occurring while escalating METH doses is likely to have a significant impact as an endogenous protective mechanism against neurodegeneration. Representative micrographs of vacuoles and the graph reporting the total number of vacuoles within controls and METH-treated cells are reported in Figure 4A and Figure 4B, respectively. Again, representative micrographs of mitochondria and the graph reporting the total number of mitochondria within controls and METH-treated cells are reported in Figure 4C and Figure 4D, respectively. When immunogold stoichiometry for HSP70 was carried out, the spontaneous increase in the protein was found to occur in the cytosol (see the graph in Supplementary Figure S1A), although the level within autophagy-like vacuoles was reduced compared with controls (see the graph in Supplementary Figure S1B) and, despite a marked cytosolic increase, no change in the level of HSP70 was measured within mitochondria (see the graph in Supplementary Figure S1C). In this calculation, it is critical to consider that the number of HSP70-positive vacuoles decreased (see the graph in Supplementary Figure S1D) and the number of HSP70-positive mitochondria is not altered (see the graph in Supplementary Figure S1E). In fact, when expressing the ratio between vacuole and cytosol HSP70, a further decrease follows METH administration (see the graph in Figure 4E). When looking at another critical subcellular area, such as mitochondria, we found a similar variation in the ratio, with a marked decrease in mitochondrial HSP70 (see the graph in Figure 4F). Thus, despite increasing in the whole cell, HSP70 moves away from the most sensitive areas in neurodegeneration. This is in line with the decrease in healthy mitochondria and the increase in the total mitochondrial mass, which depends on the accumulation of altered organelles.

**Figure 5:**
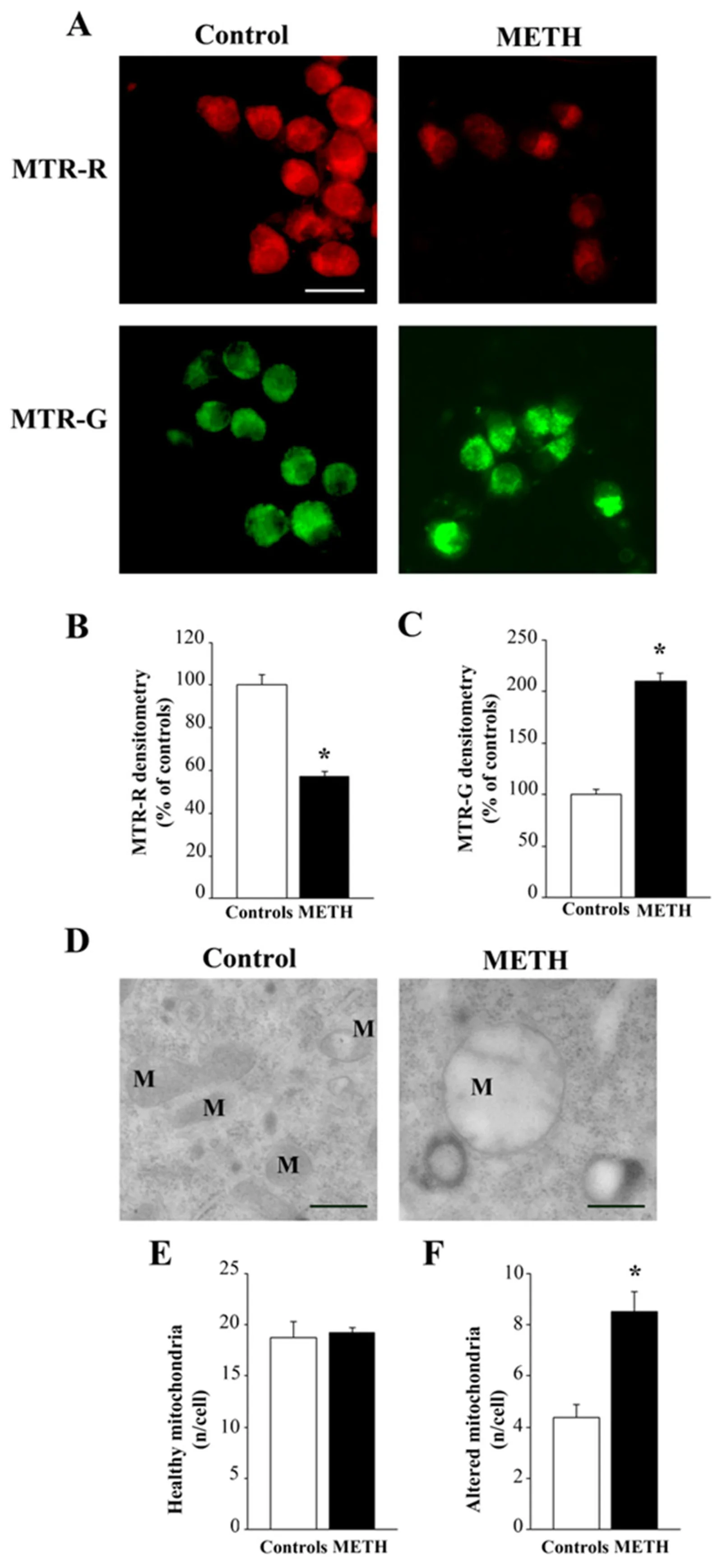
METH induces mitochondrial damage. Representative pictures in (**A**) show MTR-R and MTR-G histofluorescence, which decreased and increased, respectively, following treatment with 100 µM METH. The graphs in (**B**,**C**) report MTR-R and MTR-G densitometry. These data indicate that METH produces a loss of healthy mitochondria along with net increase in total mitochondrial mass. Representative electron micrographs in (**D**) show mitochondria from controls and METH-treated cells. The graphs in (**E**,**F**) report the number of healthy and altered mitochondria, which were counted according to the criteria reported in the methods. MTR-R and MTR-G histofluorescence was expressed as the mean percentage ± S.E.M. from *n* = 70 cells per group, assuming controls = 100%. Values related to mitochondrial ultrastructure were expressed as the mean ± S.E.M. of *n* = 50 cells per group. * *p* < 0.05 compared with controls. M = mitochondria. Scale bars (**A**) = 24 µm; (**D**) = 400 nm.

This is evident from representative pictures in Figure 5A, reporting a decrease in MTR-R and an increase in MTR-G, which indicate that METH produces a loss of healthy mitochondria and a net increase in total mitochondrial mass, which is necessarily due to the engulfment of damaged, stagnant mitochondria, as visible also from the TEM micrographs. The level of healthy mitochondria, indirectly assessed by MTR-R, is reported in the graph in Figure 5B, while the total mitochondrial mass is shown by the staining of MTR-G, which is reported in the graph in Figure 5C. Figure 5D shows representative micrographs of mitochondria in controls and METH-treated cells. The graphs in Figure 5E,F report the number of healthy and altered mitochondria, respectively, as determined by direct counting using ultrastructural morphometry, according to the criteria reported in the Methods Section. This has inherent implications for the significance of compartmentalization of HSP70 during METH toxicity.

**Figure 6:**
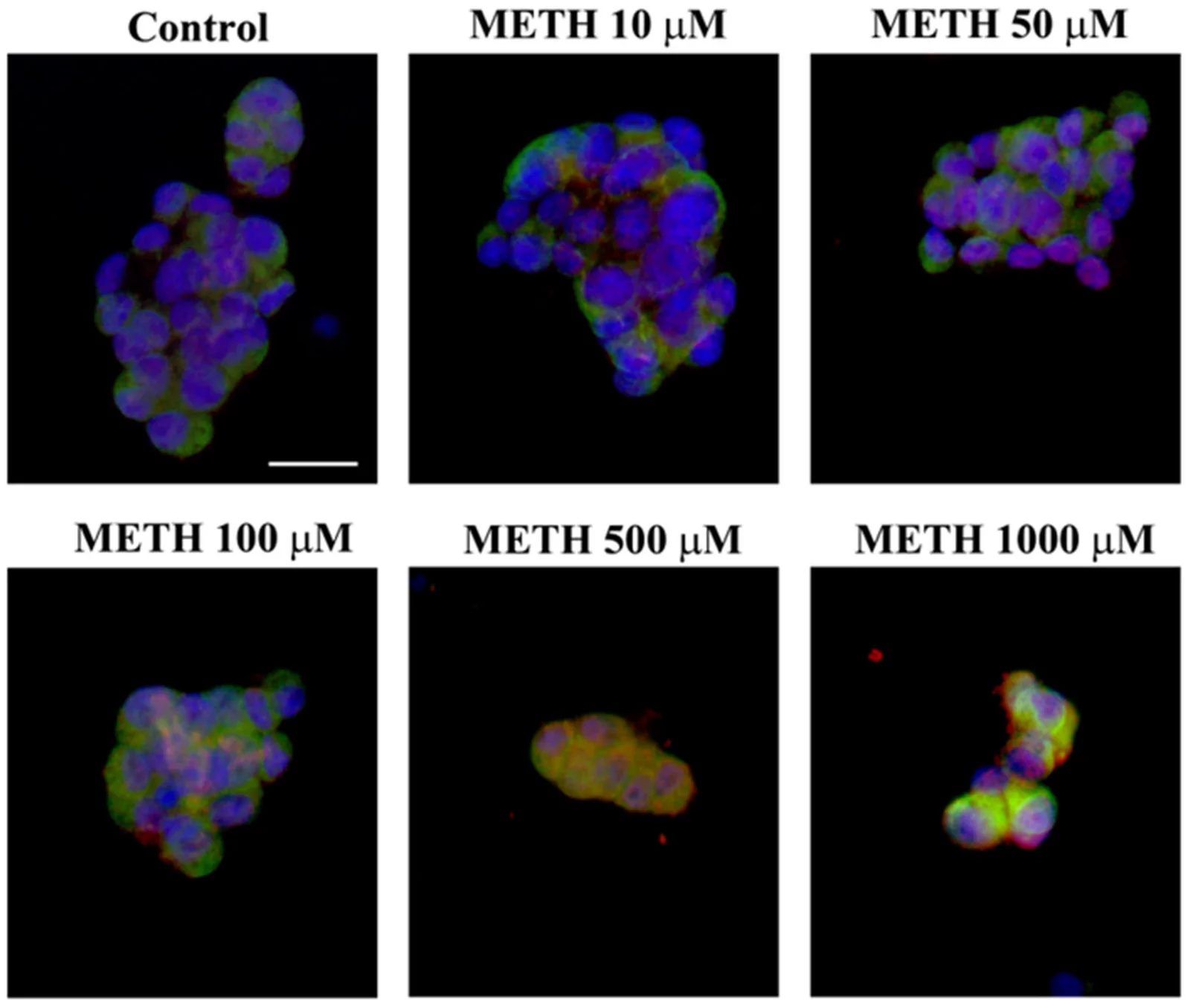
METH induces a dose-dependent co-localization of cleaved caspase 3 with HSP70. Representative pictures show cleaved caspase 3 and HSP70 immunofluorescence in controls and METH-treated cells. Most fluorescent areas for cleaved caspase 3 overlap with HSP70. Scale bar = 18 µm.

It may be hypothesized that METH exposure produces cell damage that requires a higher level of HSP70 to be counteracted. In line with this, the protein level is overexpressed, as measured in the present study, which is in line with a stimulation of HSP70 mRNA observed during METH administration [140]. Still, the persistence of METH impedes the compartmentalization of HSP70 within autophagy-like vacuoles (present study and Mastroiacovo [43]) and mitochondria, which limits the efficacy of the compensatory response. Remarkably, following these high METH doses, the fluorescent areas for cleaved caspase 3 overlap with HSP70 fluorescent areas (Figure 6), both diverging from the pattern of FJ-B histofluorescence. Since METH-induced cell death does not significantly involve apoptosis, the role of such an elevated cleaved caspase 3 has intriguing implications related to potential protective effects [136,141]. In line with this, one should not disregard that cleaved caspase 3 per se was shown to possess chaperone-like properties [139].

### 3.3. METH Induces a Dose-Dependent Increase in TRX1 That Co-Localizes with LC3-II, LAMP1, and LAMP2

**Figure 7:**
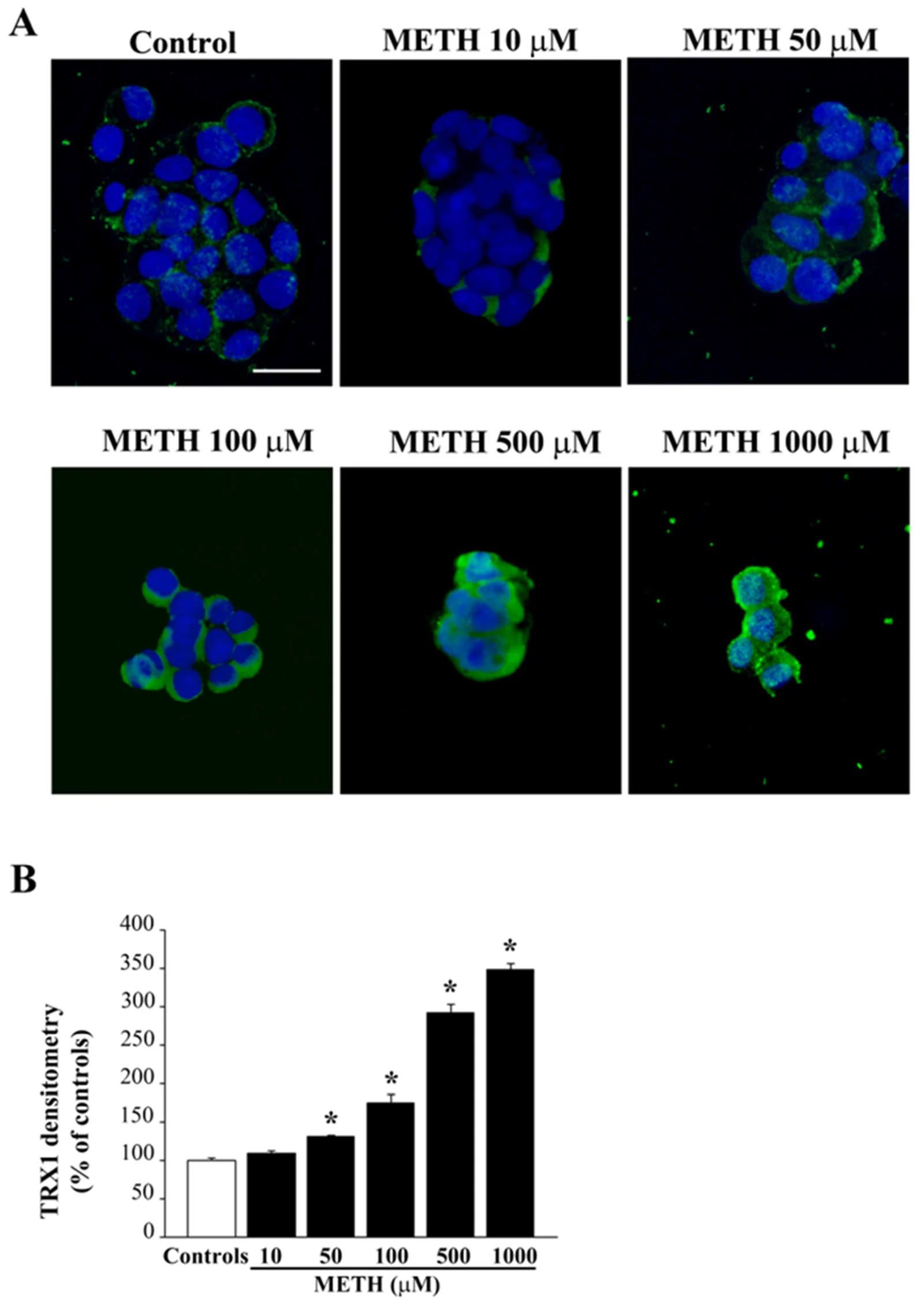
Immunofluorescence for TRX1 increases dose-dependently following METH. Representative pictures in (**A**) show the increase in TRX1 immunofluorescence produced by increasing doses of METH. Data reported in graph (**B**) represent densitometric measurements from *n* = 70 cells per group. Data are given as the mean percentage ± S.E.M., assuming controls = 100%. * *p* < 0.05 compared with controls. Scale bar (**A**) = 18 µm.

The net increase in cytosolic TRX1 under the effects of increasing METH doses is shown in Figure 7 (see the representative pictures in Figure 7A and the graph in Figure 7B). The increase in TRX1 measured by densitometry following light microscopy at the highest METH dose (1000 µM) is similar to the increase reported for HSP70 (roughly exceeding four-fold over controls).

**Figure 8:**
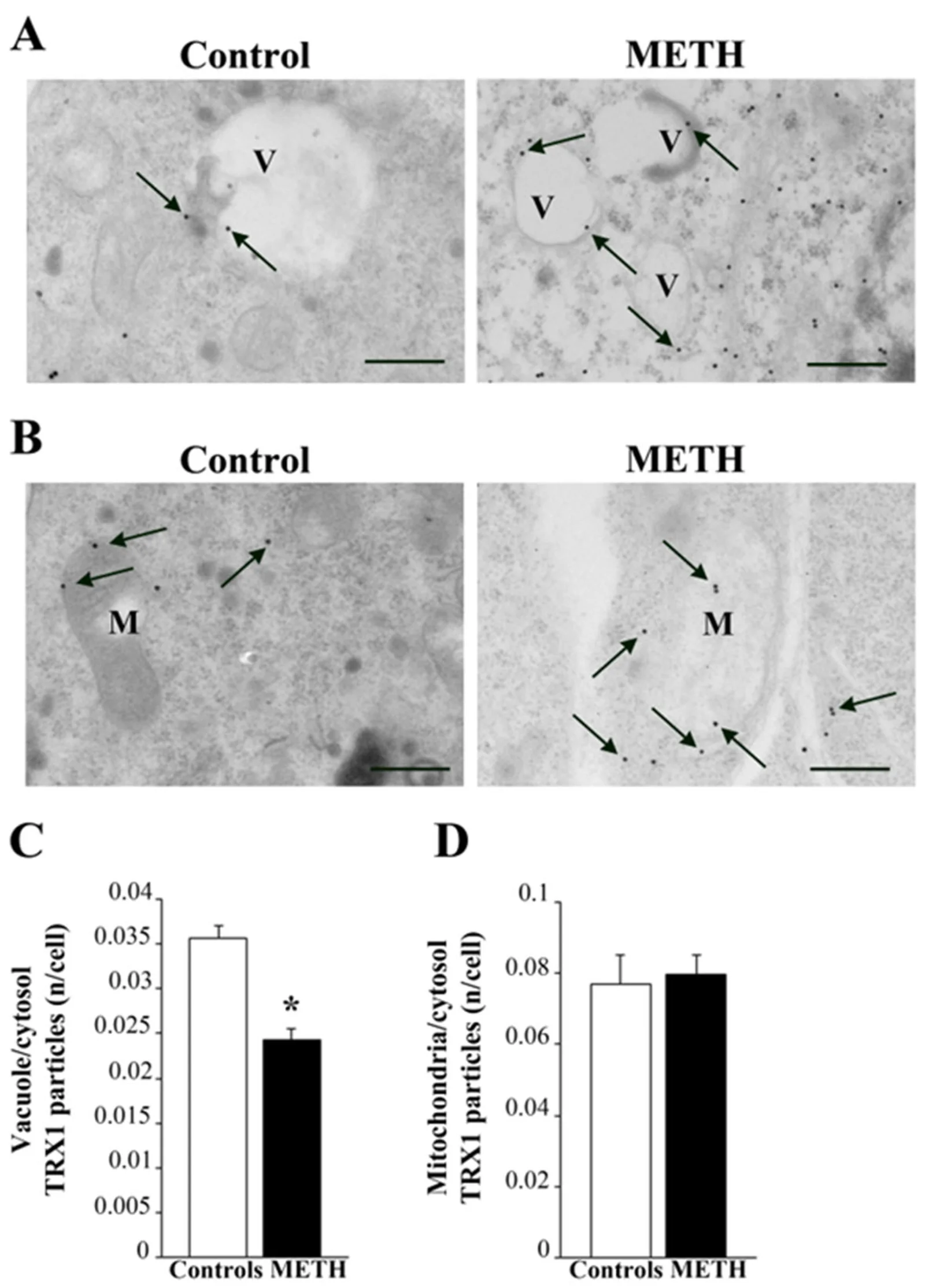
METH affects the cellular localization of TRX1. Representative micrographs show (**A**) vacuoles stained with TRX1 immunogold particles (arrows) and (**B**) mitochondria stained with TRX1 immunogold particles (arrows) within control cells and following treatment with 100 µM METH. The graphs report (**C**) the ratio of TRX1 within vacuoles to that within cytosol and (**D**) the ratio of TRX1 within mitochondria to that within cytosol. Each value represents the mean ± S.E.M. of *n* = 50 cells per group. * *p* < 0.05 compared with controls. V = vacuole; M = mitochondria. Scale bars (**A**,**B**) = 400 nm.

When this protein was measured through in situ stoichiometry by TEM (see the representative pictures in Figure 8A,B), the METH-induced increase in authentic TRX1—which was measured in the cytosol—was much more attenuated compared with HSP70, as reported in the graph in Supplementary Figure S2A. Thus, immunogold, despite confirming an increase in TRX1, as found using light microscopy, yields a substantial difference in the levels of the protein. At present, it is unknown why this may occur. It is reported that TRX1 may occur both as an independent polypeptide or it may be present as a specific domain within larger proteins. This is expected to produce a stronger signal when larger molecules are included in the sample thickness (whole cell, 10–12 µm using light microscopy compared with 70 nm using electron microscopy), which means 20-fold-thinner samples on TEM and which suppresses the chance to visualize the TRX1 domain. According to this interpretation, we may assume that the increase in TRX1 measured by TEM represents the real increase produced by METH of the sole TRX1-independent peptide. A difference between HSP70 and TRX1 following METH concerns their compartmentalization. In detail, while HSP70 within vacuoles was significantly reduced by METH (see the graph in Supplementary Figure S1B), the level of TRX1 within vacuoles remained unaltered (see the graph in Supplementary Figure S2B). Instead, the mitochondrial compartmentalization of TRX1 was markedly increased by METH (see the graph in Supplementary Figure S2C), which contrasts with the lack of effects observed for the mitochondrial level of HSP70 (see the graph in Supplementary Figure S1C). These data need to be interpreted in light of the total numbers of vacuoles and mitochondria following METH (see the graphs in Figure 4B,D): in both cases, METH increases the number of both organelles. This is internally validated by the data that TRX1-positive vacuoles increase following METH (see the graph in Supplementary Figure S2D). Similarly, an increase is measured for TRX1-positive mitochondria (Supplementary Figure S2E). All these data explain the compartmentalization data inferred by the ratio of compartmentalized TRX1 vs. cytosolic TRX1. As reported in the graph in Figure 8C, the ratio of vacuole TRX1/cytosol TRX1 was reduced by METH, which indicates that METH mostly increases the protein in the whole cytosol. In the case of mitochondria, the ratio of TRX1 in the mitochondria and cytosol is not modified by METH (see the graph in Figure 8D).

**Figure 9:**
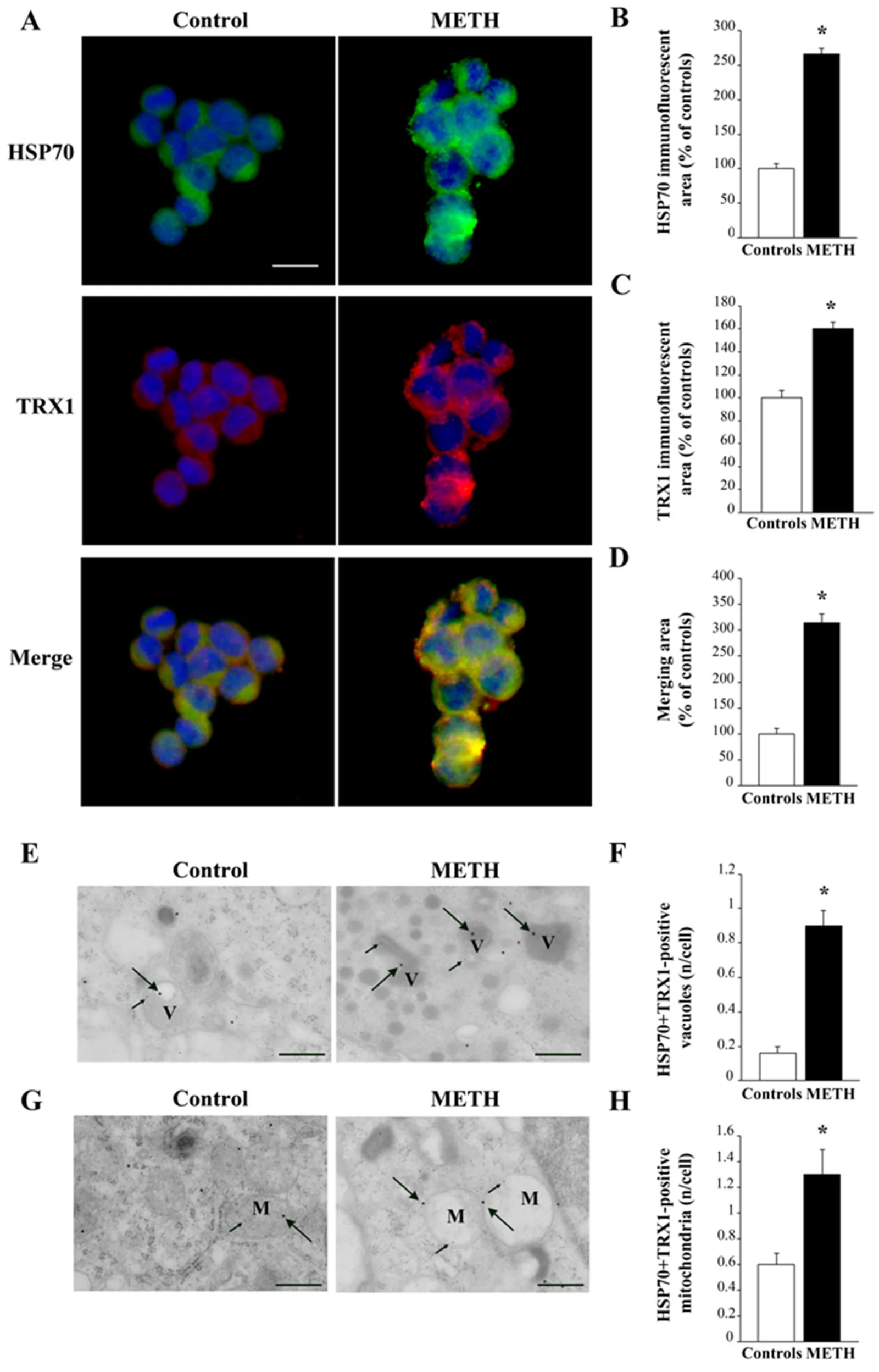
METH induces co-localization of TRX1 and HSP70. Representative pictures in (**A**) show immunofluorescence for HSP70, TRX1, and their co-localization following the dose of METH (100 µM). Graphs in (**B**–**D**) report the METH-dependent increase in immunofluorescent area for (**B**) HSP70, (**C**) TRX1, and (**D**) their co-localization. Representative micrographs in (**E**) show vacuoles co-stained with HSP70 immunogold particles (thick arrows) and TRX1 immunogold particles (thin arrows). Graph in (**F**) reports the number of vacuoles co-stained with HSP70 and TRX1. Representative micrographs in (**G**) show mitochondria co-stained with HSP70 immunogold particles (thick arrows) and TRX1 immunogold particles (thin arrows). Graph in (**H**) reports the number of mitochondria co-stained with HSP70 and TRX1. Data reported in graphs (**B**–**D**) were obtained by measuring immunofluorescent areas from *n* = 70 cells per group. Data are reported as the mean percentage ± S.E.M., assuming controls = 100%. Data reported in graphs (**F**,**H**) are expressed as the mean ± S.E.M. of *n* = 50 cells per group. * *p* < 0.05 compared with controls. V = vacuole; M = mitochondria. Scale bars (**A**) = 15 µm; (**E**,**G**) = 400 nm.

These data call for the calculation of how much HSP70 and TRX1 are co-localized within various compartments. The immunofluorescence provides the illusion that proteins are greatly co-localized (see the representative Figure 9A and the graphs in Figure 9B–D). This apparent co-localization was further dissected by ultrastructural in situ stoichiometry, showing that one protein is in the vacuoles while the other is rather peri-vacuolar following METH (see the representative micrographs in Figure 9E and the graph in Figure 9F, and representative micrographs in Figure 9G and the graph in Figure 9H).

The increase in both TRX1 and HSP70 following METH administration was further associated with the expression of some classic markers of the autophagosomes and lysosomes, such as LC3-II, LAMP1, and LAMP2.

**Figure 10:**
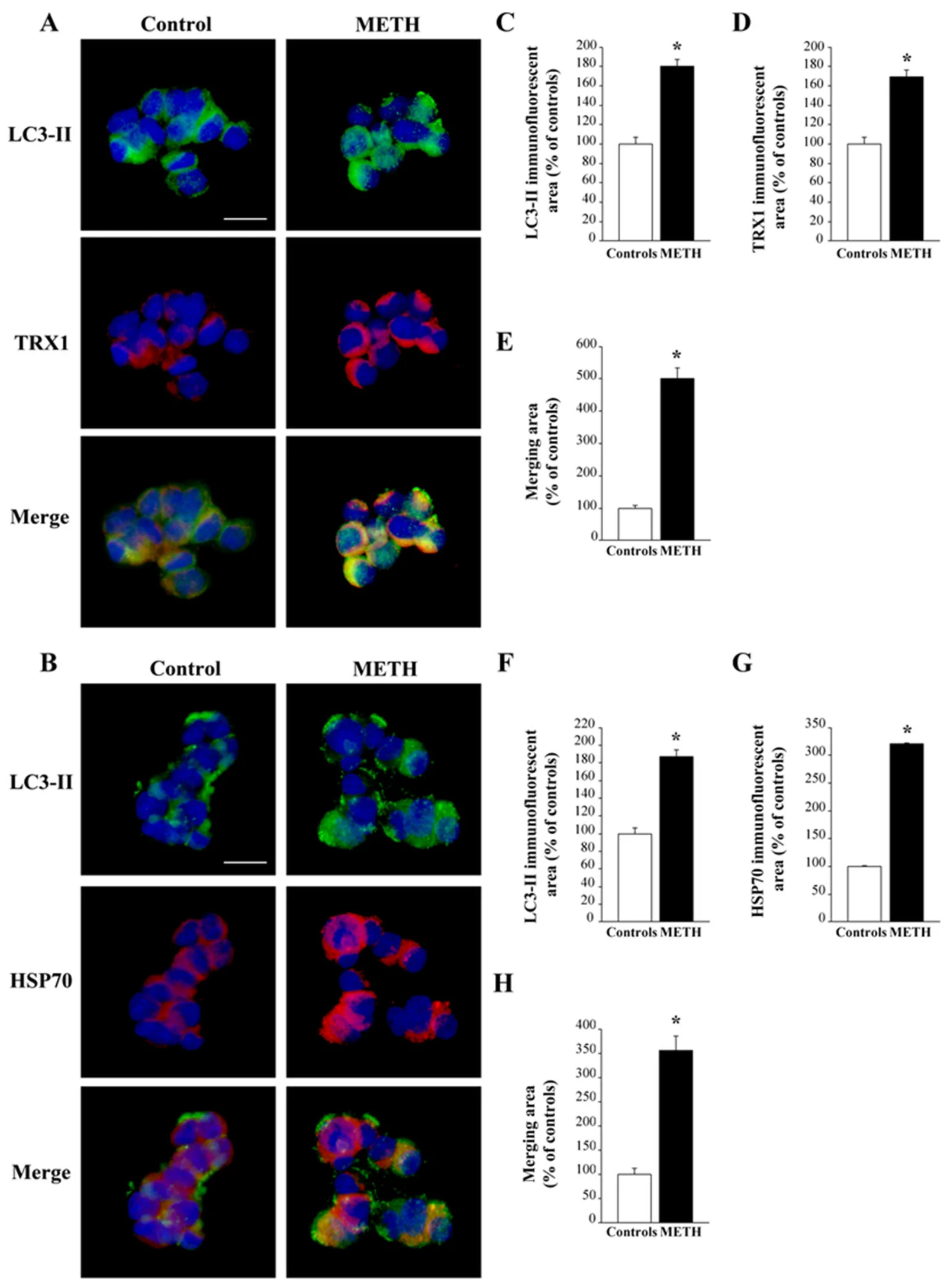
METH-induced increase in TRX1 and HSP70 overlaps with increased LC3-II. Representative pictures in (**A**) report the concomitant increase in TRX1 with the classic autophagy marker represented by the lipidated form of LC3 (LC3-II). A considerable merging of immunostaining can be appreciated. Similarly, representative pictures in (**B**) show the concomitant increase in HSP70 with LC3-II. A discrete merging is present. The fluorescent areas for these immunostainings are reported in the graphs (**C**–**H**). In detail, when the immunofluorescence for LC3-II (graph in (**C**)) and TRX1 (graph in (**D**)) are considered, the increase produced by METH is two-fold compared with controls for each antigen, while the merging rises to five-fold compared with controls (graph in (**E**)). When the immunofluorescence for LC3-II (graph in (**F**)) is compared with that of HSP70 (graph in (**G**)), the increase produced by METH is in excess of three-fold over controls (graph in (**G**)) compared with two-fold over controls for LC3-II. In the case of HSP70, the merging rises to roughly three-fold higher than controls (graph in (**H**)). Data reported in graphs (**C**–**H**) were obtained by measuring immunofluorescent areas from *n* = 70 cells per group. Data are reported as the mean percentage ± S.E.M. of controls, assuming controls = 100%. * *p* < 0.05 compared with controls. Scale bars (**A**,**B**) = 18 µm.

We found that following 100 µM METH, the increase in both TRX1 and HSP70 was concomitant with the autophagosome marker LC3 (see the representative Figure 10A,B), which was specifically stained in its lipidated, active isoform (LC3-II). The increase in LC3-II was merging much more with TRX1 compared with HSP70 (see the graph in Figure 10C–H), which suggests a specific association with autophagy activation. Again, such an increase, similar to HSP70 and TRX1, needs to be interpreted in light of a compensatory mechanism, since autophagy induction protects against, while autophagy inhibition worsens, METH toxicity [64,127,142].

**Figure 11:**
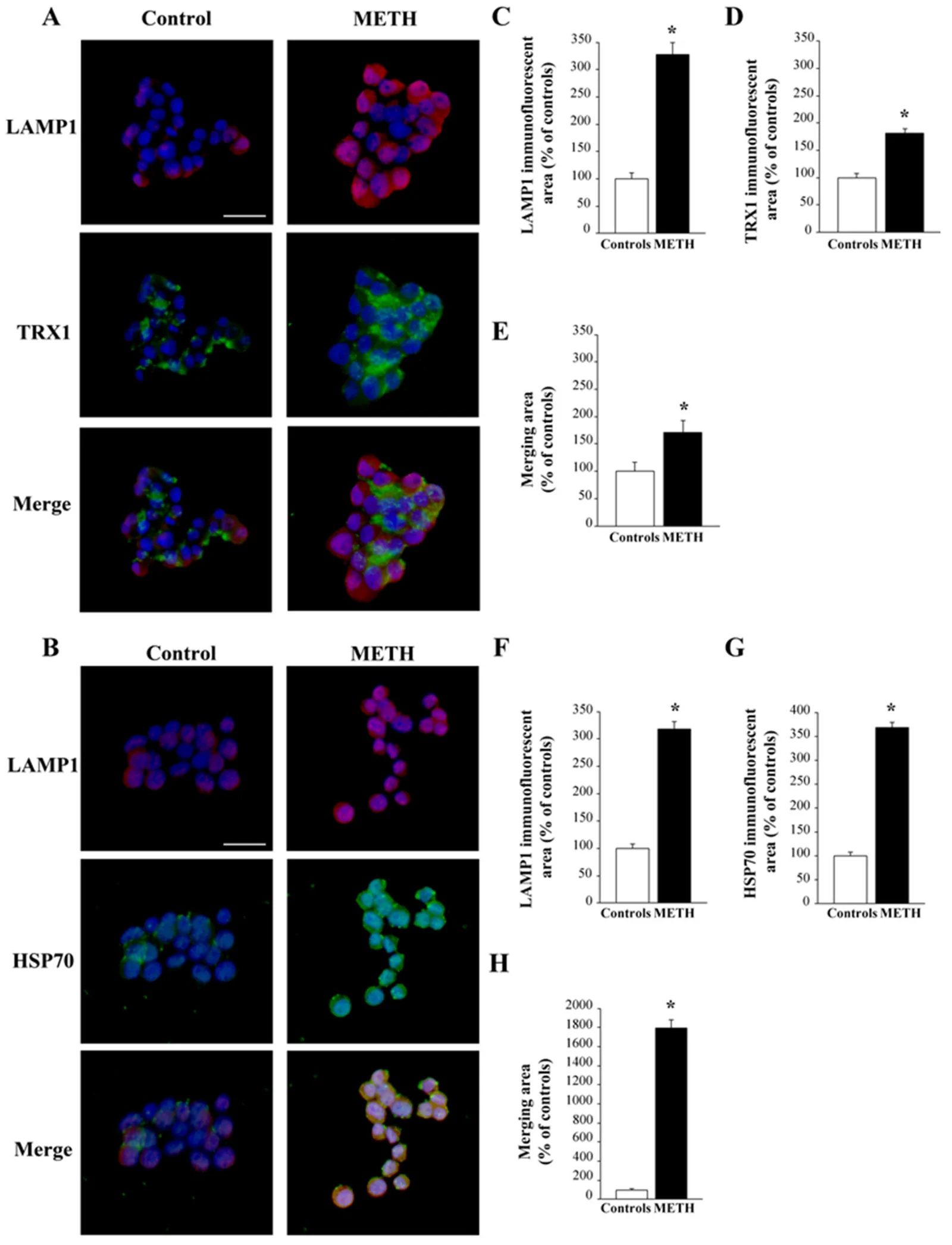
The increase in TRX1 and HSP70 produced by METH overlaps with the increase in lysosome marker LAMP1. Representative immunofluorescence for LAMP1 compared with TRX1 (**A**) or HSP70 (**B**) shows that METH increases all these markers, although to a different extent, as counted in the graphs (**C**–**H**). In detail, the increase in LAMP1 (**C**) surpasses that measured for LC3-II in Figure 10 since it is over three-fold higher than controls. The graph (**D**) reports the increase in TRX1 following METH. The merging of the immunofluorescent staining of LAMP1 and TRX1 (**E**) is the lowest and is less than two-fold compared with controls. The increase in LAMP1 reported in the graph in (**F**) is comparable to the increase in HSP70 (**G**). Remarkably, the merging of LAMP1 and HSP70 reaches eighteen-fold compared with controls (**H**). Data reported in graphs (**C**–**H**) were obtained by measuring immunofluorescent areas from *n* = 70 cells per group. Data are reported as the mean percentage ± S.E.M. of controls, assuming controls = 100%. * *p* < 0.05 compared with controls. Scale bars (**A**,**B**) = 30 µm.

Similarly, the lysosome marker LAMP1 was increased following METH (see the representative Figure 11A,B). The merging of LAMP1 was preferentially measured for HSP70 compared with TRX1 (see the graphs in Figure 11C–H). This suggests a co-localization of HSP70 occurring preferentially with the lysosomal compartment.

**Figure 12:**
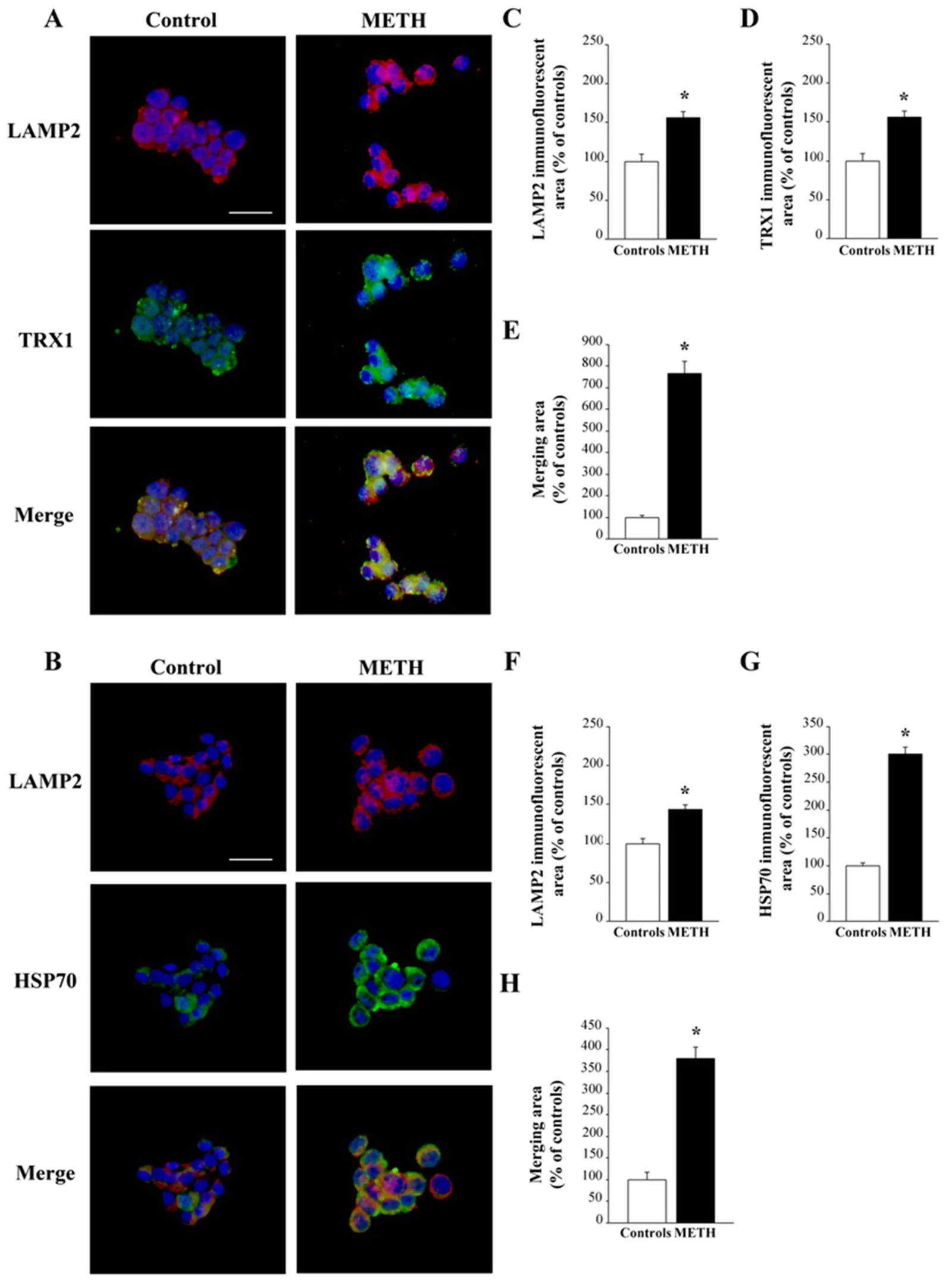
The increase in TRX1 and HSP70 produced by METH overlaps with the CMA marker LAMP2. Representative pictures in (**A**,**B**) show the staining of the CMA marker LAMP2 and its merging with TRX1 and HSP70. Although the increase in METH-induced immunofluorescence for either LAMP1 (**C**) or TRX1 (**D**) was moderate (less than two-fold of controls), the amount of merging induced by METH (**E**) was impressive (roughly seven-fold of controls). In the case of HSP70, although the increase in LAMP2 (**F**) or HSP70 (**G**) induced by METH surpasses that measured for TRX1, the merging of HSP70 with LAMP2 induced by METH (**H**) was less than 20% of the increase in the merging measured for TRX1 and LAMP2 (**E**). Data reported in graphs (**C**–**H**) were obtained by measuring immunofluorescent areas from *n* = 70 cells per group. Data are reported as the mean percentage ± S.E.M. of controls, assuming controls = 100%. * *p* < 0.05 compared with controls. Scale bars (**A**,**B**) = 30 µm.

Since HSPs act as chaperones, the merging with the CMA marker is an interesting issue to look for. As displayed in the representative pictures in Figure 12A,B, the merging of TRX1 with LAMP2 was in excess compared with HSP70, which confirms the association of TRX1 with chaperone-mediated autophagy (CMA). This is shown in the graphs in Figure 12C–H. In summary, the merging of LAMP1 and HSP70 reaches eighteen-fold compared with controls. This suggests that the increase in HSP70 preferentially recruits the lysosome compartment with a specificity index of 9, considering the ratio of the METH-induced increase in the LAMP1-positive lysosome compartment compared with the METH-induced increase in the whole cells (averaging various cell domains).

In order to assess the authentic effect of 100 µM METH on autophagic activity, autophagic flux was measured and immunofluorescence intensity for LC3-II and p62 was measured within the same cells. The representative pictures in Supplementary Figure S3A show that, as expected for an autophagy inhibitor, in bafilomycin A1-treated cells, the immunofluorescence for both LC3-II and p62 increased compared with control cells. This was also confirmed by the graphs in Supplementary Figure S3B,C, reporting the optical density for HSP70 and p62 immunofluorescence, respectively. In detail, bafilomycin A1 is a potent inhibitor of the vacuolar-type H+ ATPase, leading to lysosomal impairment and inhibition of the late stages of autophagy [143]. Thus, degradation of LC3-II and p62 is blocked by bafilomycin A1, and both proteins accumulate within the cell. In METH-treated cells, immunofluorescence for LC3-II and p62 is increased similarly to that measured in bafilomycin A1 (see the Supplementary Figure S3A and the graphs in Supplementary Figure S3B,C). This is consistent with a METH-induced impairment of autophagy, due to: (i) the cargo of misfolded proteins produced by METH [39,142,144,145,146,147], which overwhelms the autophagy machinery; (ii) the METH-induced delocalization of LC3-II and p62 from the vacuoles to the cytosol, which de-potentiates the autophagy machinery and impairs the autophagy progression [39,125,126,142]. This leads to the accumulation of both LC3-II and p62 proteins, which further increase following the combined treatment with METH and bafilomycin A1 (Supplementary Figure S3).

## 4. Discussion

The present study indicates that two different proteins, which interact as chaperones (HSP70 and TRX1), are strongly induced following exposure to METH. Both proteins occur under baseline conditions in the whole cytosol, being clustered within autophagy-like vacuoles and mitochondria.

This study further investigates whether METH-induced cytopathology alters the expression of HSP70 and TRX1 since both proteins exert a neuroprotective effect against METH toxicity [40,41,99]. The study demonstrates that METH increases dose-dependently the levels of both HSP70 and TRX1.

The levels of these proteins following METH were found to be in excess compared with control conditions. These data are consistent across light and electron microscopy. These findings connecting HSPs with METH abuse and damage replicate data in humans from Kitamura [148] and Ondruschka et al. [149] following 50 autopsies. Similarly, Madea et al. [69] reported on 39 autopsies of humans who abused amphetamines, describing an increase in HSP70. These studies found an increase in HSP70 within brain and peripheral organs in persons abusing METH. The mechanism for such an increase was hypothesized to depend on METH-induced hyperthermia [69]. Remarkably, the present study, carried out in catecholamine cells in vitro, definitively rules out a role for hyperthermia in inducing both HSP70 and TRX1. Rather, the increase in these proteins measured in the present study, as in humans who abuse METH, is likely to reflect the compensatory empowerment of cell-clearing systems to counteract METH-induced toxicity. In fact, (i) overexpression of these proteins confers protection against METH toxicity; (ii) they are recruited by autophagy; and (iii) overexpression of autophagy protects against METH toxicity. The toxicity induced by METH was established in the present study by a dose–response curve, ranging from 10 µM up to 1000 µM.

This range of METH doses fits with most of the established literature [145,150,151,152], and corresponds to the range of METH doses administered in the intercellular space (biophase) in in vivo experiments [153,154,155,156].

We found that higher METH doses alter cell shape and size, producing irregular, eosinophilic areas surrounded by pale vesicle-like domains, which are reminiscent of the tubulo-vesicular structures developing in the course of neurodegeneration [125]. These areas were recently investigated by CLEM as pale eosinophilic, non-electron-dense cell domains that recruit components of the autophagolysosome pathway with several damaged mitochondria, high concentrations of p62, and the small heat-shock protein ubiquitin, along with some amount of alpha-synuclein [49,50]. The histochemical staining with H&E allows for counting the dose-dependent decrease in cell number. These data are replicated here using FJ-B as a specific degeneration marker [157]. These methods confirm that METH administration causes dose-dependent cell degeneration starting at 50 µM. It is remarkable that measurement of cell degeneration using caspase 3 diverges from FJ-B, suggesting that the significance of caspase 3 goes beyond the staining of degenerating areas. Remarkably, following high METH doses, fluorescent areas for cleaved caspase 3 overlap with HSP70 fluorescent areas, both diverging from the pattern of FJ-B histofluorescence. Since METH-induced cell death does not significantly involve apoptosis, the role of such an elevated cleaved caspase 3 assumes intriguing implications related to potential protective effects [136,141]. In line with this, one should not disregard that cleaved caspase 3 per se was shown to possess chaperone-like properties [139]. In fact, in the present study, the staining with caspase 3 bridges the expression of HSP70 as well as the concomitant expression of HSP70 and TRX1 and their merging.

In detail, increasing doses of METH-triggered a concomitant increase in HSP70, which is known to protect against METH toxicity [41]. This indicates that METH administration triggers an increase in HSP70 as a compensatory effect.

Since silencing HSP70 worsens METH toxicity, and overexpression of HSP70 protects against METH toxicity [41], the progressive increase in the endogenous protein is likely to exert protective mechanisms against neurodegeneration. When immunogold stoichiometry for HSP70 was carried out, the spontaneous increase in the protein was found to occur in the cytosol, while the level within autophagy-like vacuoles was reduced compared with controls. When looking at another critical subcellular area, such as mitochondria, a marked decrease in HSP70-stained mitochondria was detected. Thus, despite increasing in the whole cell, HSP70 moves away from degeneration-sensitive cell areas to spread randomly in the cytosol.

This is consistent with a decrease in MTR-R and an increase in MTR-G, which indicate a loss of healthy mitochondria despite a net increase in total mitochondrial mass, which is due to the increase in damaged, stagnant mitochondria confirmed by TEM. It may be hypothesized that METH exposure produces cell damage that requires a higher level of HSP70 to be counteracted. This is in line with a stimulation of HSP70 mRNA found during METH administration [140]. Still, the persistence of METH impedes the compartmentalization of HSP70 within autophagy-like vacuoles (present study and Mastroiacovo et al. [43]) and mitochondria, which limits the efficacy of the compensatory response. This is also confirmed by the measure of autophagic flux, which is impaired following METH.

Similarly, the present study indicates that METH produces a dose-dependent increase in TRX1, as measured by whole-cell densitometry following light microscopy. Such an increase is similar to that measured for HSP70. Nonetheless, when the METH-induced increase in authentic TRX1 was measured in the cytosol by immunogold stoichiometry, this was less pronounced compared with HSP70. At present, such a discrepancy cannot be explained. A further difference between HSP70 and TRX1 following METH concerns their compartmentalization, since HSP70 within vacuoles was significantly reduced by METH, while the level of TRX1 within vacuoles remains unaltered. In contrast, the level of TRX1 molecules within mitochondria increased following METH.

**Figure 13:**
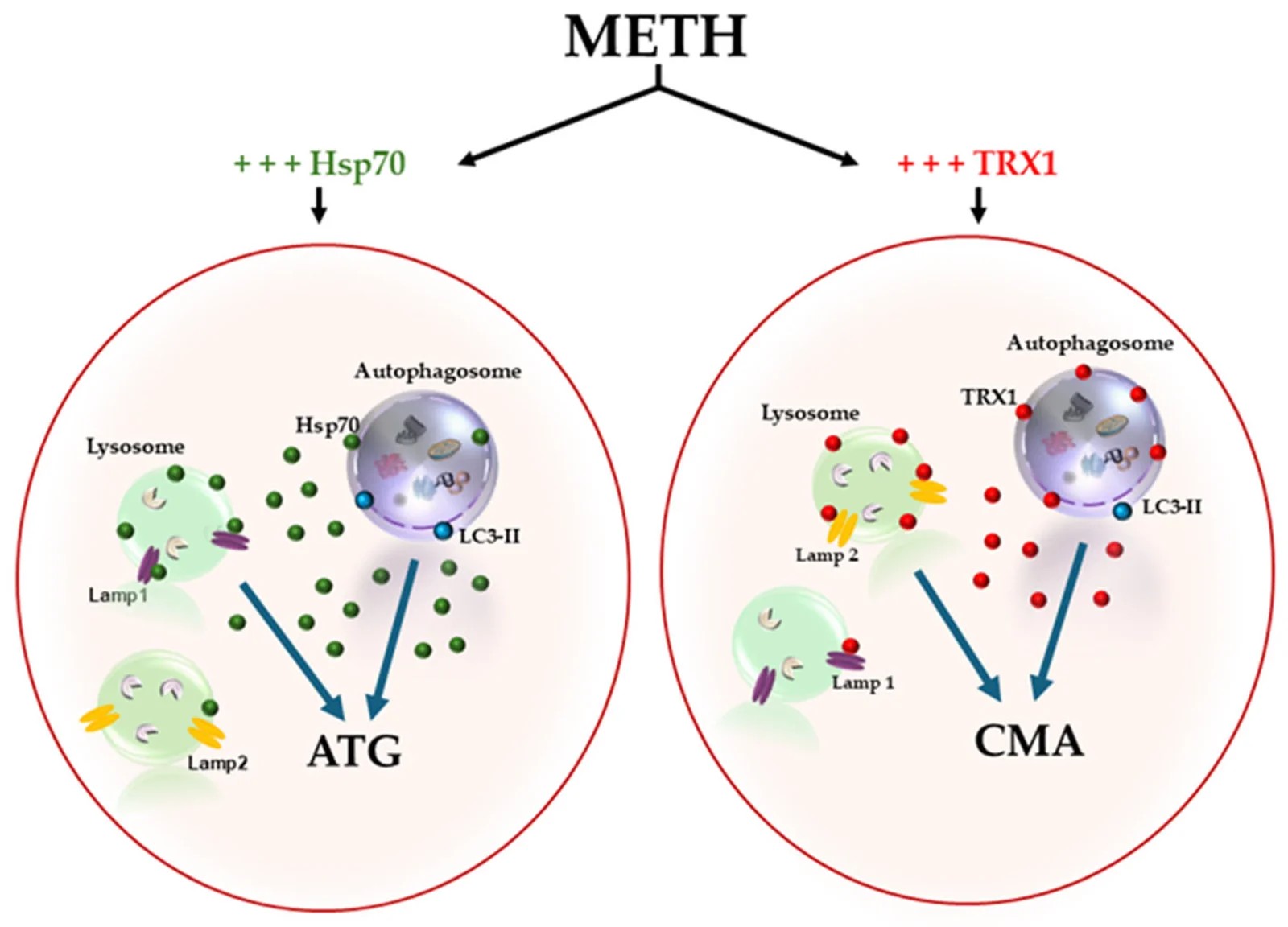
METH-induced HSP70 and TRX1 segregate within autophagosomes and lysosomes. The increase in HSP70 is concomitant with the increase in the autophagosome marker LC3-II and the lysosome marker LAMP1, which are involved in autophagy (**left side** of the figure). Similarly, TRX1 increases concomitantly with the autophagosome marker LC3-II, while it is preferentially associated with the lysosome marker LAMP2, which is involved in chaperone-mediated autophagy (**right side** of the figure). ATG = autophagy; CMA = chaperone-mediated autophagy.

The increase in both TRX1 and HSP70 following METH administration is associated with the expression of some classic markers of the autophagosomes and lysosomes, such as LC3-II, LAMP1, and LAMP2 (Figure 13).

We found that, following 100 µM METH, the increases in both TRX1 and HSP70 were concomitant with the autophagosome marker LC3, which was specifically stained in its lipidated, active isoform (LC3-II). The increase in LC3-II was merging much more with TRX1 compared with HSP70, which suggests a specific association with autophagy activation (Figure 13). Again, such an increase needs to be interpreted in light of a compensatory mechanism since autophagy induction protects, while autophagy inhibition worsens, METH toxicity [64,127,142]. Similarly, the lysosome marker LAMP1 increased following METH. The merging of LAMP1 was preferentially measured for HSP70 compared with TRX1. Since HSPs act as chaperones, the merging with the CMA marker is an interesting issue to look for. The merging of TRX1 with LAMP2 was in excess compared with HSP70, which confirms the association of TRX1 with CMA (Figure 13).

The results of the present study indicate that HSP70 and TRX1 increased within specific cell compartments involved in neurodegeneration.

The cell compartments where these proteins increase correspond to those involved in stress response [158,159], which is in line with the historical progression of our understanding of the role of HSPs in neurodegeneration. In detail, heating was the first noxious stimulus to be identified among a variety of detrimental factors that need to be cleared by an effective autophagolysosome system. In this way, the specific decrease in these proteins within autophagosomes following METH confirms the data we reported ex vivo following brain ischemia [43]. Thus, HSPs appear to be a part of the compensatory apparatus relying on cell clearance to counteract both acute and chronic insults, including neurotoxicity induced by a drug of abuse. Interestingly, the synthesis of these proteins is not limited to extreme, stressful conditions, and it occurs even in baseline conditions when additional roles are played by the vesicles involved in the stress response. This is the case of recycling and modulating neurotransmitter release [160]. It is noteworthy that HSPs represent an excess of 1% of total protein content [54], which calls for multiple roles and functions of this protein class. In recent years, the name chaperone or co-chaperone was often used to indicate the ability of HSPs to regulate the folding of other proteins and to interact with cell proteins to regulate their shape and appropriate degradation [70,73]. These actions also apply to caspase 3 [139]. Similarly, the primary identification of ubiquitin in the class of HSPs [54,55] confirms the seminal role of these proteins in baseline conditions to regulate protein recycling and degradation. Therefore, it is not surprising that, just like ubiquitin, they need to interact with misfolded proteins through an E3 ubiquitin ligase such as parkin; this is also the case for HSP70, which needs to interact properly with parkin in order to prevent neurodegeneration, such as that occurring in PD [31]. In line with this, a mutation of parkin produces inherited parkinsonism [161], while alterations of TRX1 are described in the substantia nigra of patients suffering from PD as well as in experimental PD models [93,162,163,164,165,166,167], and HSP70 is a constant component of Lewy bodies [28,168,169].

Therefore, one may expect that mutations of classic HSPs may sort PD as well [29,170,171]. In detail, this may concern mutations in HSP70 [172]. Similarly, parkinsonism induced by a loss of function is expected to be compensated by an increased activity of HSP70, either induced genetically or pharmacologically [173]. The other protein investigated in the manuscript, TRX1, is expected to possess analogous associations [174,175]. On the other hand, downregulation of glutaredoxin (which belongs to the TRX family, a group of redox proteins that also include TRX1 and glutathione dehydroascorbate reductase [176,177,178,179,180]) leads to degeneration of nigral dopamine-containing neurons [174,181,182,183].

### Limitations of the Study

Despite the evidence provided, the relevance of molecular interaction between HSP70 and TRX1 remains poorly investigated. Specific constructs should be tested analogous to those that are supposed to occur spontaneously to assess the enhancement of neuroprotection. Similarly, the protective effects of cleaved caspase 3, which may act as a co-chaperone on the efficacy of the autophagic pathway, should be deeply characterized along with the activity of HSP70 as a protein recruited by the autophagy machinery.

## Data Availability

The raw data supporting the conclusions of this article will be made available by the authors upon request.

## Supplementary Materials

The following supporting information can be downloaded at: https://www.mdpi.com/article/10.3390/biomedicines14092134/s1, Figure S1. METH (100 µM) alters cellular placement of HSP70; Figure S2. METH (100 µM) affects the cellular placement of TRX1; Figure S3. METH impairs autophagy flux.

## Abbreviations

The following abbreviations are used in this manuscript:

AD	Alzheimer’s disease
CMA	Chaperone-mediated autophagy
CLEM	Combined light and electron microscopy
FJ-B	Fluoro-Jade B
H&E	Hematoxylin & Eosin
HSP70	Heat-shock protein 70
LAMP1	Lysosomal-associated membrane protein 1
LAMP2	Lysosomal-associated membrane protein 2
LC3	Microtubule-associated protein 1A/1B-light chain 3
LC3-II	Lipidated LC3
METH	Methamphetamine
MTR-G	MitoTracker green
MTR-R	MitoTracker red
PD	Parkinson’s disease
TRX1	Thioredoxin-1
