# Supplementary material for: Endogenous Expression of HSP70 and Thioredoxin-1 During Methamphetamine-Induced Degeneration
Source: Biomedicines. 2026 Sep 21;14(9):2134. doi: 10.3390/biomedicines14092134 (PMC13604769; doi:10.3390/biomedicines14092134)
Supplement: Supplementary file 1 [file biomedicines-14-02134-s001.zip › biomedicines-4466052-supplementary.pdf]

Supplementary Figures

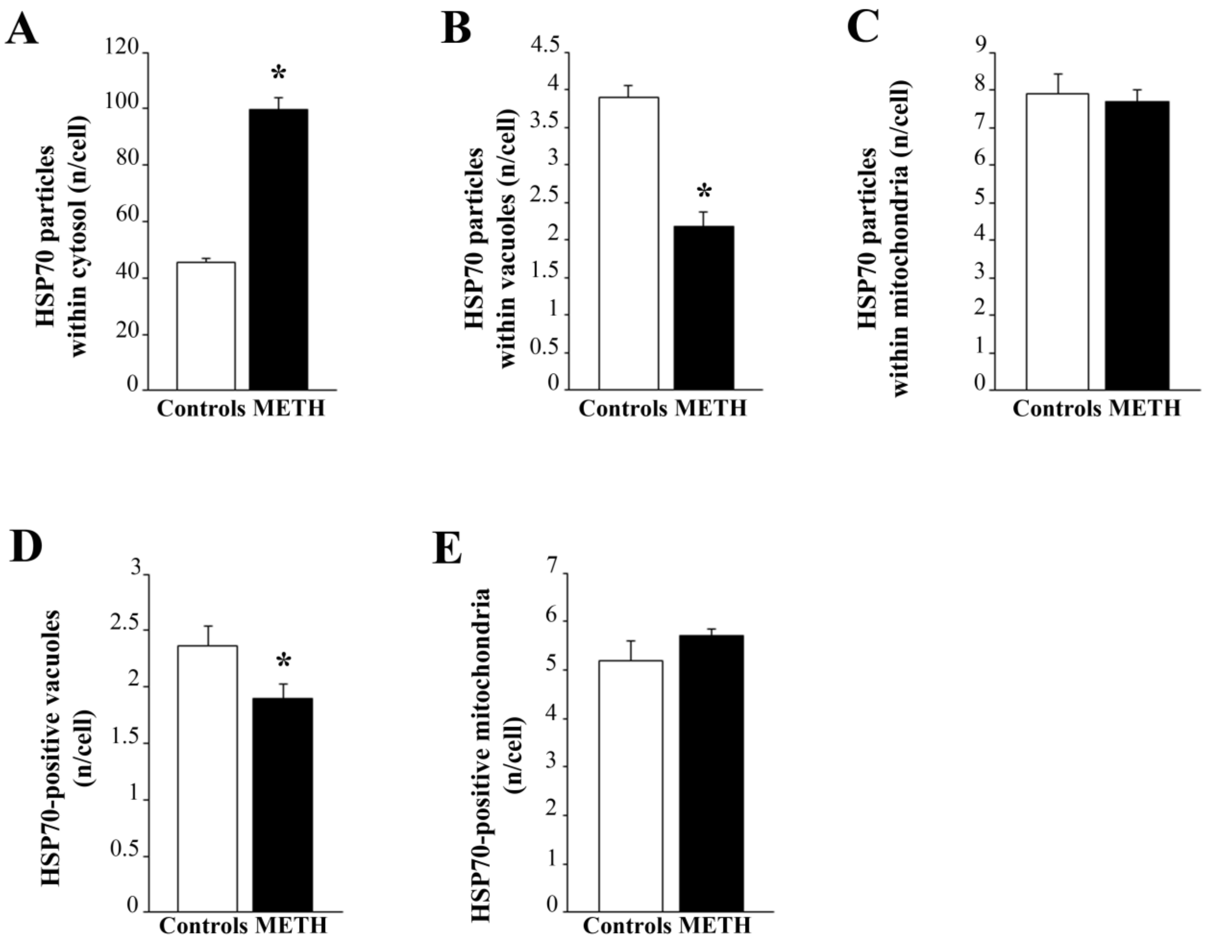

Supplementary Figure S1. METH (100 μM) alters cellular placement of HSP70. The graphs report: (**A**) the number of immunogold particles for HSP70 within the cytosol; (**B**) the number of immunogold particles for HSP70 within autophagy-like vacuoles; (**C**) the number of immunogold particles for HSP70 within mitochondria; (**D**) the number of HSP70 positive vacuoles; (**E**) the number of HSP70 positive mitochondria. Each value represents the mean±S.E.M. of n=50 cells per group. *P<0.05 compared with controls.

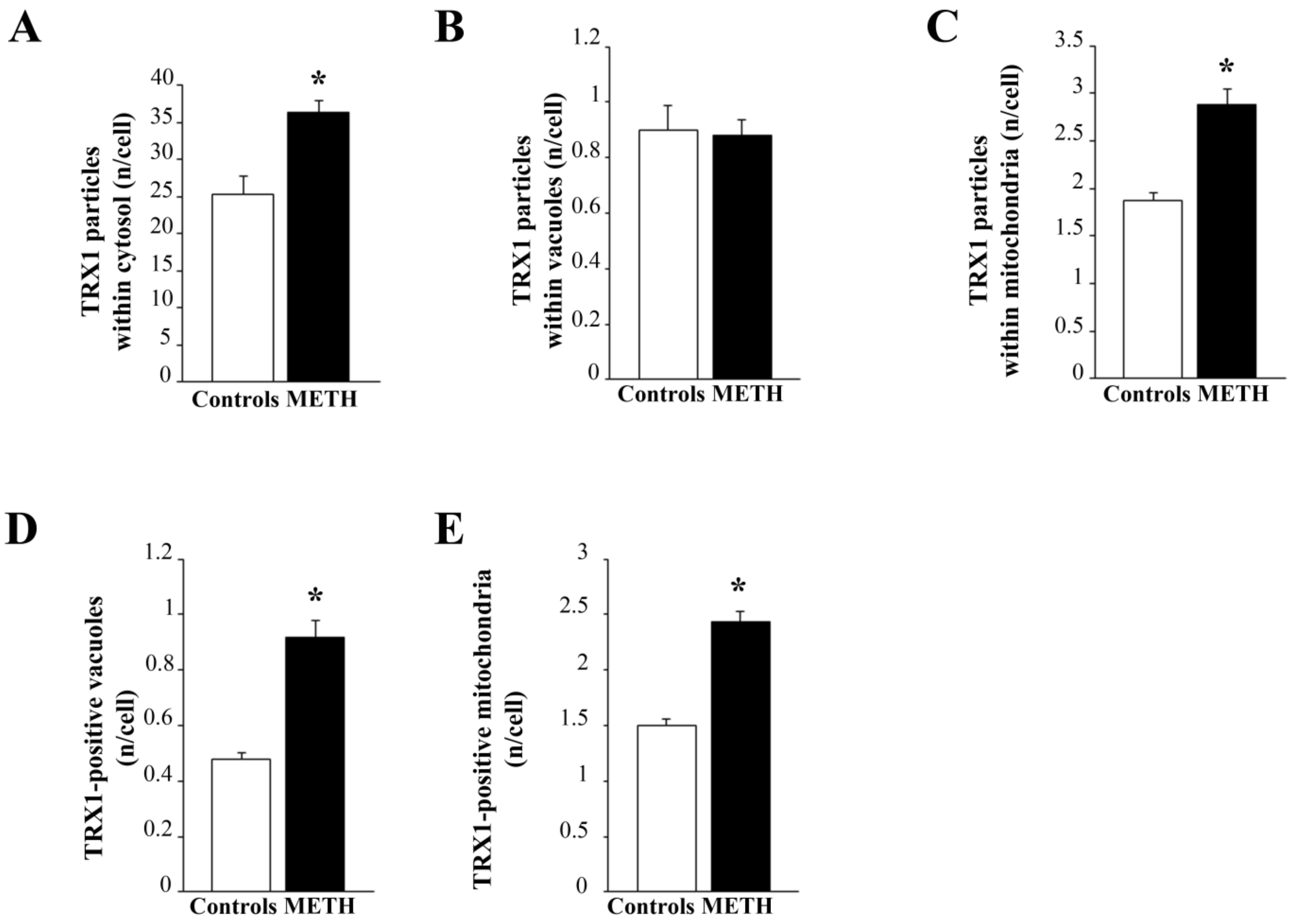

Supplementary Figure S2. METH (100 μM) affects the cellular placement of TRX1. The graphs report: (**A**) the number of immunogold particles for TRX1 within the cytosol; (**B**) the number of immunogold particles for TRX1 within autophagy-like vacuoles; (**C**) the number of immunogold particles for TRX1 within mitochondria; (**D**) the number of TRX1-positive vacuoles; (**E**) the number of TRX1-positive mitochondria. Each value represents the mean±S.E.M. of n=50 cells per group. *P<0.05 compared with controls.

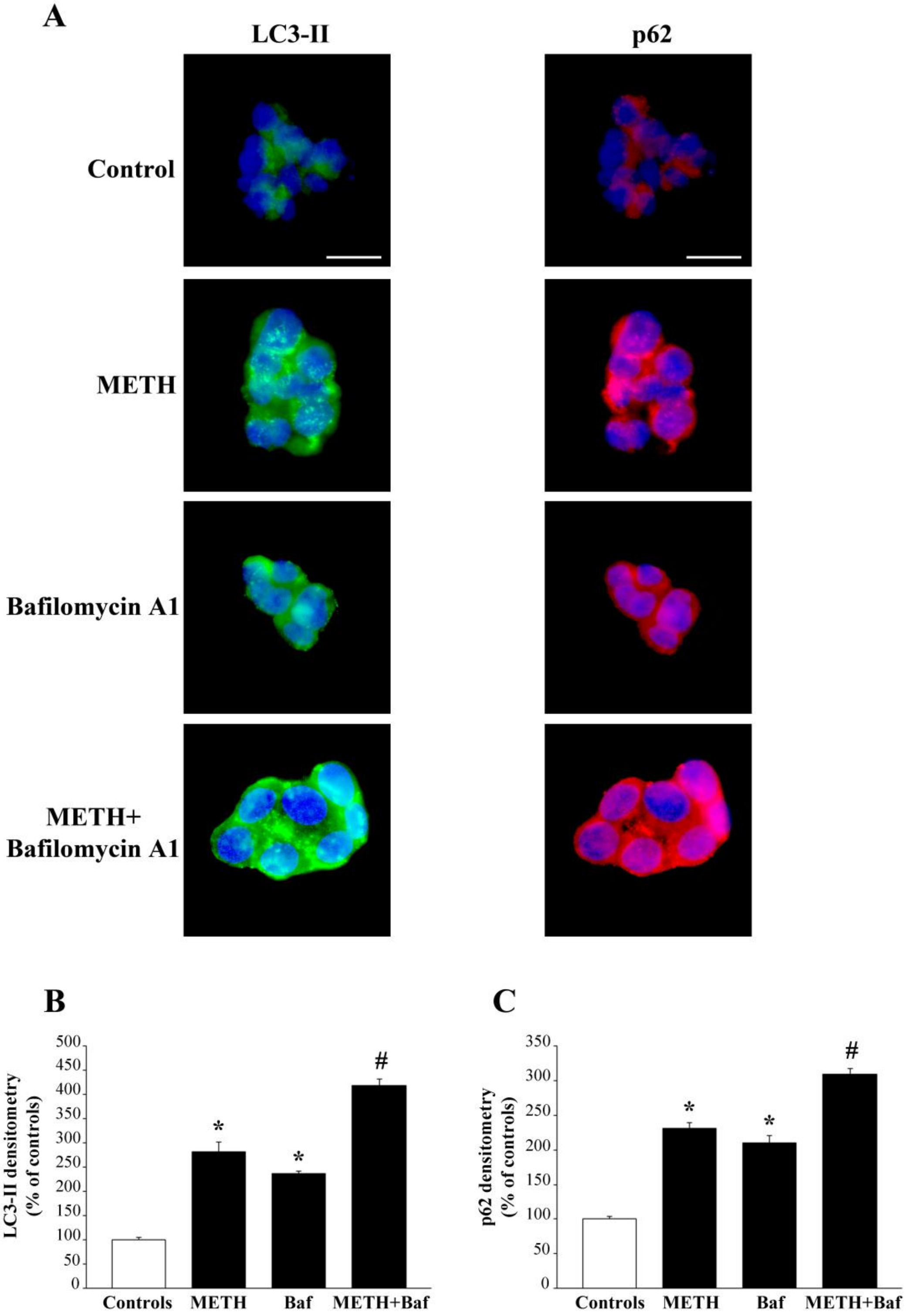

Supplementary Figure S3. METH impairs autophagy flux. (**A**) Immunofluorescence for LC3-II and p62 was carried out within the very same cells and representative pictures from control cells, and cells treated with METH 100 μM, bafilomycin A1 100 nM, or combined METH and

bafilomycin A1 are shown. An increased immunofluorescence for both the proteins is evident in METH-treated cells compared with controls. Such an increase is comparable to that produced by the autophagy inhibitor bafilomycin A1. These findings are confirmed by the graphs in (**B**) and (**C**), which report the densitometry of LC3-II and p62, respectively, measured in n=70 cells per group. Data are reported as the mean percentage±S.E.M. of controls, assuming controls=100%. Baf=bafilomycin A1; METH=methamphetamine. *P<0.05 compared with controls; #P<0.05 compared with other groups. Scale bars: 17 µm.
